# Association Between Postoperative Pneumonia and Prognosis in Patients With Non‐Traumatic Subarachnoid Hemorrhage: An Analysis of the MIMIC‐IV Database

**DOI:** 10.1002/brb3.71916

**Published:** 2026-11-09

**Authors:** Hao Wang, Taotao Shi, Tao Ge, Hongcai Wang, Maosong Chen

**Affiliations:** ^1^ Department of Neurosurgery The Affiliated Lihuili Hospital of Ningbo University Ningbo Zhejiang People's Republic of China

**Keywords:** length of stay, postoperative pneumonia, prognosis, subarachnoid hemorrhage

## Abstract

**Objective:**

To investigate the association between postoperative pneumonia (POP) and prognosis in patients with nontraumatic subarachnoid hemorrhage (SAH).

**Methods:**

This retrospective cohort study used the MIMIC‐IV database. Adult SAH patients who underwent surgical treatment with postoperative sedation were included, excluding traumatic SAH, nonsurgical cases, ICU stay ≤ 1 day, and those without sedative use. Patients were divided into the POP and non‐POP groups. Multivariable logistic regression identified independent risk factors for POP, validated by LASSO regression and random forest analysis. A progressive adjustment strategy evaluated POP's association with in‐hospital, 30‐day, 90‐day, and 1‐year mortality, and length of stay (LOS). Sensitivity analyses included inverse probability of treatment weighting (IPTW)/doubly robust analysis, short‐stay exclusion, survivor‐restricted analysis, and Fine‐Gray competing risk models. Mortality at 30 days, 90 days, and 1 year was ascertained only among patients who survived to hospital discharge and is therefore reported as post‐discharge mortality.

**Results:**

Among 505 included patients, 189 (37.4%) developed POP. Factors independently associated with higher POP odds included WFNS Grades IV–V (OR = 2.373, *p* < 0.001), dexmedetomidine use (OR = 2.026, *p* = 0.001), and COPD (OR = 1.817, *p* = 0.039). Higher admission potassium (OR = 0.526, *p* = 0.010) and acetaminophen use (OR = 0.597, *p* = 0.012) were independently associated with lower POP odds. POP was not independently associated with in‐hospital mortality (OR = 1.030, *p* = 0.908) but significantly prolonged ICU LOS (ratio = 1.651, *p* < 0.001) and total hospital LOS (ratio = 1.765, *p* < 0.001). LOS findings were consistent across all sensitivity analyses, while in‐hospital mortality results were less stable across analytical frameworks and should be interpreted cautiously. Post‐discharge mortality among patients who survived to hospital discharge was 7.5% versus 3.7% at 30 days, 11.6% versus 8.2% at 90 days, and 18.5% versus 11.0% at 1 year.

**Conclusions:**

In surgical nontraumatic SAH patients, POP correlated with prolonged ICU and hospital LOS. High WFNS grade, dexmedetomidine use, and COPD were independently associated with increased POP odds. These findings highlight the potential value of POP prevention strategies to reduce LOS and healthcare resource consumption.

## Graphical abstract

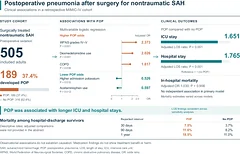

## 1 Introduction

Subarachnoid hemorrhage (SAH) is a severe cerebrovascular event characterized by bleeding into the subarachnoid space, accounting for approximately 5%–7% of all strokes (de Rooij et al. 2007; Etminan et al. 2019). Although the incidence of SAH is relatively low (global age‐standardized incidence rate of approximately 9.1 per 100,000 person‐years), its case fatality rate and morbidity are exceedingly high, with in‐hospital mortality reaching 20%–40% (Connolly et al. 2012; de Rooij et al. 2007; Lawton and Vates 2017). In recent years, advances in endovascular interventional therapy and microsurgical techniques have improved acute‐phase outcomes for SAH patients; however, postoperative complications remain a major determinant of prognosis (Wartenberg and Mayer 2006; Wartenberg et al. 2006).

Postoperative pneumonia (POP) is one of the most common infectious complications following SAH surgery, with reported incidence rates ranging from 10% to 30% (Jin et al. 2023; Li et al. 2024; Yuan et al. 2022). SAH patients are particularly vulnerable to POP due to multiple factors, including impaired cough reflex and airway protection resulting from altered consciousness, mechanical ventilation use, prolonged bed rest, and immunosuppression (Solenski et al. 1995; Wartenberg and Mayer 2006). Previous studies have demonstrated that medical complications, such as fever, anemia, hyperglycemia, and pneumonia, can significantly affect SAH patient outcomes (Diringer et al. 2011; Wartenberg et al. 2006). Wartenberg et al. (2006) found that medical complications following SAH surgery were significantly associated with poor functional outcomes and mortality at 3 months. Frontera et al. (2008) reported that nosocomial infectious complications increased the risk of death and prolonged hospitalization in SAH patients.

However, existing research on the relationship between POP and long‐term prognosis in SAH patients remains insufficient. Most studies have focused on constructing predictive models for pneumonia risk factors (Jin et al. 2023; Li et al. 2024; Yuan et al. 2022), while the independent impact of pneumonia occurrence on mortality (particularly long‐term mortality) and length of stay (LOS) has not been systematically evaluated. Moreover, given that POP is a time‐varying event occurring during hospitalization, methodological considerations such as time‐dependent bias (immortal time bias) and competing risks warrant careful attention. Of note, prior MIMIC‐IV‐based studies (Jin et al. 2023; Li et al. 2024) have concentrated predominantly on constructing predictive models for POP risk in aneurysmal SAH, without systematically addressing the independent causal impact of POP on multi‐timepoint mortality outcomes. Critically, those investigations did not employ causal inference frameworks—such as propensity score weighting or competing risk models—to disentangle the effect of POP from the prognostic burden of the underlying disease. The present study directly addresses these methodological gaps by integrating progressive adjustment strategies, IPTW/doubly robust analysis, and Fine‐Gray competing risk models to rigorously evaluate both the occurrence determinants and the prognostic consequences of POP. Regarding potential cohort overlap: Wang et al. (2025) used the MIMIC‐IV database to examine anesthetics and POP risk in non‐traumatic SAH, and Jin et al. (2023) and Li et al. (2024) similarly analyzed POP risk factors in MIMIC‐IV SAH cohorts. Our cohort was defined by stricter eligibility criteria (mandatory postoperative sedation use, ICU stay > 1 day, surgical treatment), yielding *n* = 505, which likely represents a partially overlapping but distinct patient subset. The incremental contribution of the present study lies in its focus on the prognostic consequences of POP rather than its prediction, and in its application of causal inference methods (IPTW/doubly robust analysis, Fine‐Gray competing risk models) not employed in prior MIMIC‐IV POP‐in‐SAH work.

Therefore, this study aimed to utilize the MIMIC‐IV large‐scale critical care database to: (1) systematically identify independent risk factors for POP in nontraumatic SAH patients who underwent surgery and received postoperative sedation; (2) evaluate the independent association of POP with mortality at different time points and LOS using a progressive adjustment strategy; and (3) assess the robustness of conclusions through multiple sensitivity analysis approaches, including IPTW/doubly robust analysis and competing risk models, to provide evidence‐based guidance for POP prevention and management strategies in SAH patients.

## 2 Methods

### 2.1 Study Design and Data Source

This was a retrospective cohort study based on the MIMIC‐IV database. The MIMIC‐IV database contains clinical data from ICU patients at Beth Israel Deaconess Medical Center (BIDMC) in Boston, USA, including demographic characteristics, vital signs, laboratory tests, medication records, diagnostic codes, surgical records, and prognostic information. This study was approved by the relevant institutional review board and was conducted in compliance with the MIMIC‐IV data use agreement.

### 2.2 Study Population and Eligibility Criteria

This study included adult patients diagnosed with SAH via ICD codes in the MIMIC‐IV database. Inclusion criteria were: (1) age ≥ 18 years; (2) confirmed SAH diagnosis by ICD codes; (3) surgical treatment during hospitalization; (4) postoperative sedative use (at least one of midazolam, dexmedetomidine, or propofol); and (5) ICU stay > 1 day. Exclusion criteria were: (1) traumatic SAH with concomitant cranial trauma; (2) no surgical treatment; (3) no postoperative sedative use; and (4) ICU stay ≤ 1 day. The term nontraumatic SAH was adopted in this study because patient identification relied on ICD codes, which distinguish traumatic from nontraumatic etiology but do not systematically differentiate aneurysmal from other nontraumatic causes (e.g., perimesencephalic, arteriovenous malformation). This coding‐based classification aligns with the terminology used in prior MIMIC‐IV SAH studies. Where “spontaneous SAH” is used in the neurological literature, it is largely synonymous with nontraumatic SAH in the context of administrative database research.

Initial screening identified 2808 SAH patients by ICD codes. Sequential exclusions removed 53 patients with cranial trauma (remaining 2755), 1057 nonsurgical patients (remaining 1698), 1150 patients without sedative use (remaining 548), and 43 patients with ICU stay ≤ 1 day (remaining 505), yielding a final cohort of 505 patients for analysis. The patient selection process is illustrated in Figure 1 (STROBE‐compliant flowchart).

**FIGURE 1:**
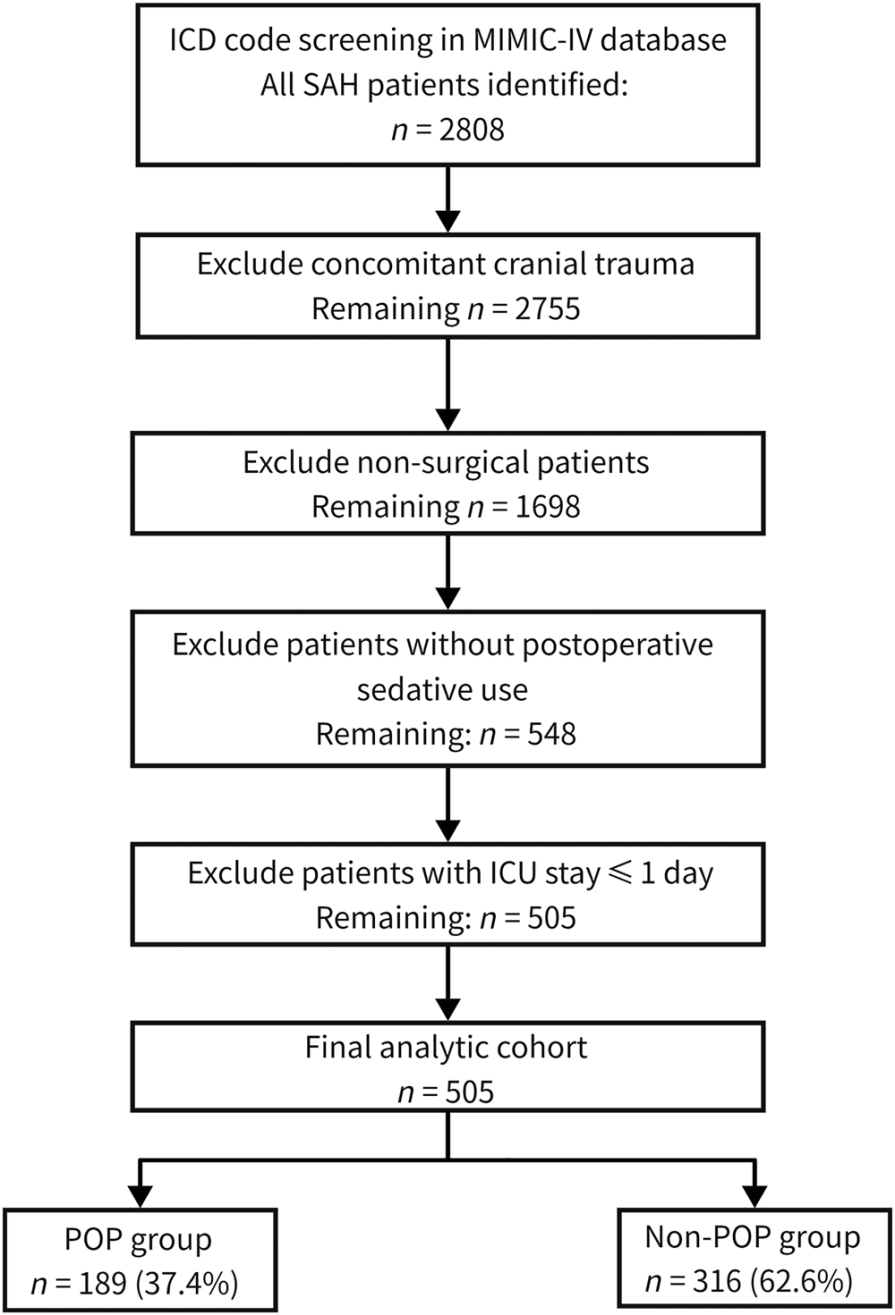
STROBE‐compliant patient selection flowchart. Initial screening identified 2808 patients with SAH by ICD codes in the MIMIC‐IV database. Sequential exclusions were performed, yielding a final analytic cohort of 505 patients (POP group *n* = 189; non‐POP group *n* = 316). ICD, International Classification of Diseases; ICU, intensive care unit; POP, postoperative pneumonia; SAH, subarachnoid hemorrhage.

### 2.3 Data Collection and Variable Definitions

POP was diagnosed according to the ICD codes (Wang et al. 2025). COPD was identified based on ICD codes for chronic obstructive pulmonary disease (COPD) in the MIMIC‐IV diagnoses_icd table, reflecting pre‐existing (not newly diagnosed) comorbidity as recorded in administrative data. It should be acknowledged that ICD‐based POP diagnosis lacks microbiological confirmation and imaging granularity; consequently, ventilator‐associated pneumonia (VAP) and nonventilator‐associated pneumonia cannot be distinguished within this dataset, which represents an inherent limitation of administrative database research. Respiratory culture results were available for the subset of patients in whom specimens were clinically obtained; these data were not used to define POP. The primary outcome was in‐hospital mortality; secondary outcomes included 30‐day mortality, 90‐day mortality, 1‐year mortality, ICU LOS, and total hospital LOS. In‐hospital death and post‐discharge death were recorded as mutually exclusive endpoints: patients who died before discharge were not counted in the 30‐day, 90‐day, or 1‐year mortality figures, which were therefore ascertained among patients who survived to hospital discharge. In the mortality models, in‐hospital deaths were coded as non‐events at follow‐up, so the follow‐up mortality estimates should be read as post‐discharge mortality among hospital survivors; a survivor‐restricted analysis is reported alongside the main estimates.

Based on clinical relevance and literature review, the following variables were collected: (1) Demographics: age, sex; (2) vital signs (first values within 24 h of admission): heart rate, systolic blood pressure (SBP), diastolic blood pressure (DBP), pulse oxygen saturation (SpO2); (3) laboratory parameters (first values within 24 h of admission): red blood cell distribution width (RDW), hemoglobin, glucose, chloride, white blood cell count (WBC), platelet count, creatinine, international normalized ratio (INR), prothrombin time (PT), partial thromboplastin time (PTT), sodium (first measurement), potassium, bicarbonate; (4) disease severity scores: Glasgow Coma Scale (GCS), World Federation of Neurosurgical Societies (WFNS) Grade (I–III coded as 1, IV–V coded as 2), Simplified Acute Physiology Score II (SAPS II), Acute Physiology Score III (APS III); (5) Charlson Comorbidity Index (CCI) and individual comorbidities: including COPD, congestive heart failure (CHF), diabetes, hyperlipidemia, coronary heart disease (CHD), cerebral ischemia, and renal disease (all based on ICD codes); (6) Treatment‐related variables: mechanical ventilation use, surgical approach (endovascular intervention vs. other), sedative use (propofol, midazolam, dexmedetomidine), and acetaminophen use. In MIMIC‐IV, WFNS grade is not natively recorded. In this study, WFNS grade was derived from the admission GCS score combined with the presence or absence of focal neurological deficits documented in the clinical notes: WFNS Grade I was defined as GCS 15 without focal deficit; Grade II as GCS 13–14 without focal deficit; Grade III as GCS 13–14 with focal deficit; Grade IV as GCS 7–12; and Grade V as GCS 3–6. This operationalization is consistent with published MIMIC‐IV‐based SAH studies (Wang et al. 2025).

Microbiological data were obtained from the MIMIC‐IV microbiology events table, which records specimen type, organism identification, and antibiotic susceptibility results. Respiratory specimens were defined as sputum, bronchoalveolar lavage, mini‐bronchoalveolar lavage, bronchial washings, and tracheal aspirate; screening specimens (e.g., nasal MRSA surveillance cultures) and entries flagged as canceled, negative, or nonspecific were excluded. Because a single specimen can yield several organisms, the analysis was based on unique isolates defined by specimen identifier, organism, and isolate number, and patient‐level summaries used the first respiratory isolate recovered during the index hospitalization, ranked by collection time and then by isolate number. Oxacillin susceptibility was used to classify *Staphylococcus aureus* isolates as methicillin‐resistant or methicillin‐susceptible. The 505 patients in the analytic cohort were linked to these microbiology records by patient identifier.

### 2.4 Statistical Analysis

Patients were grouped according to the occurrence of POP. Continuous variables were expressed as median (interquartile range) (M [IQR]) and compared using the Mann–Whitney *U* test (non‐parametric tests were uniformly applied given the typically skewed distribution of clinical data). Categorical variables were expressed as frequencies (percentages) (*n* [%]) and compared using chi‐square tests or Fisher's exact test.

#### 2.4.1 Risk Factor Analysis for POP

Univariable logistic regression was performed for each candidate variable to screen potential risk factors for POP. Variables with *p* < 0.1 were included as candidates for multivariable analysis. Multivariable model construction followed these principles: (1) age and sex were forced into the model as a priori confounders regardless of univariable results; (2) based on collinearity diagnostics (correlation matrix and variance inflation factor [VIF]), GCS and WFNS grade were highly correlated (*r* = −0.79), and WFNS grade was retained for its greater specificity to SAH; APS III was highly correlated with SAPS II (*r* = 0.74), and both were excluded from the primary model due to collinearity with WFNS grade; (3) deep vein thrombosis (DVT) and urinary tract infection (UTI) were excluded as postoperative complications rather than risk factors, to avoid reverse causation. Multicollinearity was assessed using VIF (> 5 indicating multicollinearity), and model goodness‐of‐fit was evaluated using the Hosmer–Lemeshow test. Results were reported as odds ratios (OR) with 95% confidence intervals (CI). The proportions of missing values for key laboratory variables were as follows: albumin 38.0%, neutrophil differential 38.4%, lymphocyte differential 37.2%, monocyte differential 28.3%, SBP/DBP 25.7%, and BMI 62.0%. Variables with high missingness (BMI, differential cell counts) were excluded from the primary multivariable model. For variables with low‐to‐moderate missingness (< 5%: RDW 1.4%, glucose 1.4%, PTT 2.4%, sodium 1.4%–3.6%, potassium 1.0%), a complete‐case analysis was applied; sensitivity analyses confirmed that the primary findings were robust to this approach. All analyses were conducted on the complete‐case sample (*n* = 505 for the primary model; *n* = 478 and *n* = 391 for secondary models as previously reported). In addition, a calibration plot was generated to visually assess model calibration.

LASSO regression and random forest analysis were used to validate the robustness of the traditional logistic regression results. LASSO regression used 10‐fold cross‐validation to select the optimal regularization parameter lambda, with the area under the receiver operating characteristic curve (AUC) as the evaluation metric. The random forest model was configured with 500 trees, a maximum depth of 10, and a minimum of 20 samples for splitting, with 10‐fold cross‐validation for performance evaluation. Continuous variables were standardized before LASSO analysis.

#### 2.4.2 Association of POP with Outcomes

A progressive adjustment strategy was employed to evaluate the association of POP with each mortality outcome. Four progressively adjusted logistic regression models were constructed: Model 1 (unadjusted), Model 2 (adjusted for age and sex), Model 3 (adjusted for age, sex, WFNS grade, and SAPS II), and Model 4 (adjusted for age, sex, WFNS grade, SAPS II, mechanical ventilation, CCI, diabetes, and COPD). For LOS outcomes (ICU LOS, total hospital LOS), log‐linear regression models were used (natural logarithm transformation of LOS), with results reported as ratios representing the fold change in LOS for the POP group relative to the non‐POP group.

#### 2.4.3 Sensitivity Analyses

Since the exact time of the onset of POP cannot be confirmed, there may be an immortal time between the SAH surgery and the onset of POP. To address concerns regarding time‐dependent bias and immortal time bias, three categories of feasible sensitivity analyses were conducted: (1) IPTW/doubly robust analysis: propensity scores were constructed using age, sex, WFNS grade, SAPS II, CCI, diabetes, COPD, and surgery category, followed by stabilized inverse probability weighting; doubly robust outcome models were then constructed in the weighted sample to evaluate in‐hospital mortality, ICU LOS, and total hospital LOS; (2) Short‐stay exclusion analysis: analyses were repeated after restricting to total hospital LOS ≥ 3, ≥ 5, and ≥ 7 days using multivariable logistic or log‐linear regression; and (3) Survivor‐restricted LOS analysis: among in‐hospital survivors only, ICU LOS and total hospital LOS were re‐evaluated using log‐linear regression. Covariate balance before and after IPTW was assessed using standardized mean differences (SMD). We acknowledge that these sensitivity analyses mitigate, but do not fully eliminate, the core immortal time / time‐dependent bias, since POP onset timing remains unknown and POP is treated as a fixed baseline exposure. Patients with longer hospital stays inherently have more opportunity to both develop POP and accrue LOS. A landmark analysis or time‐varying Cox formulation would more rigorously address this; however, the absence of POP onset timestamps in the current dataset precluded such analyses. This represents an important methodological limitation that should be considered when interpreting the LOS findings.

To further distinguish “in‐hospital death” and “alive discharge” as competing outcomes, a Fine‐Gray subdistribution hazard model was applied for competing risk analysis. This was implemented using the survival package in R 4.5.3. Using total hospital LOS as the time variable, two sets of models were constructed: (1) with in‐hospital death as the target event and alive discharge as the competing event; and (2) with alive discharge as the target event and in‐hospital death as the competing event. Both sets were adjusted for POP, age, sex, WFNS grade, SAPS II, mechanical ventilation, CCI, diabetes, and COPD. Cumulative incidence functions (CIF) for in‐hospital death and alive discharge were estimated at 7, 14, and 28 days. For the primary multivariable logistic regression and progressive adjustment analyses, R version 4.5.3 was used with the base stats package. LASSO regression was performed using the glmnet package (version 4.1‐8) with 10‐fold cross‐validation. Random forest analysis was conducted using the randomForest package (version 4.7‐1.2) with 500 trees; 10‐fold cross‐validation was applied for performance evaluation, and nested cross‐validation was not used, which may introduce optimism in the reported AUC estimates—this is acknowledged as a limitation. All statistical analyses were performed in R version 4.5.3.

## 3 Results

### 3.1 Baseline Characteristics

A total of 505 nontraumatic SAH patients who underwent surgery and received postoperative sedation were included. Among them, 189 (37.4%) developed POP (POP group), and 316 (62.6%) did not (non‐POP group). Baseline characteristics are presented in Table 1. Regarding demographics, the two groups were comparable in age (62.0 [49.0–71.0] vs. 59.0 [48.0–72.2] years, *p* = 0.384) and sex distribution (male: 58.7% vs. 55.1%, *p* = 0.477).

**TABLE 1:** Baseline characteristics and outcomes of patients.

Variable	Total (*n *= 505)	POP (*n *= 189)	Non‐POP (*n *= 316)	*p*
Age, years	60.0 (48.0–72.0)	62.0 (49.0–71.0)	59.0 (48.0–72.2)	0.384
Heart rate, bpm	66.0 (58.0–77.0)	68.5 (59.0–79.0)	65.0 (57.0–75.2)	0.024
SBP, mmHg	126.0 (112.0–140.0)	126.0 (112.0–139.2)	127.0 (112.0–141.0)	0.803
DBP, mmHg	63.0 (54.0–72.0)	62.0 (54.0–71.0)	63.0 (55.0–73.0)	0.369
RDW, %	13.7 (13.0–14.8)	13.9 (13.2–15.1)	13.6 (13.0–14.7)	0.051
Hemoglobin, g/dL	11.8 (10.3–13.2)	11.6 (10.1–13.1)	11.9 (10.5–13.4)	0.179
Glucose, mg/dL	133.0 (111.0–170.8)	135.0 (113.0–178.0)	131.0 (110.0–165.0)	0.238
Chloride, mEq/L	104.0 (101.0–108.0)	104.0 (101.0–108.0)	105.0 (102.0–108.0)	0.398
WBC, K/µL	11.4 (8.9–14.4)	11.9 (9.4–14.9)	11.3 (8.7–14.0)	0.158
Platelet, K/µL	186.0 (140.0–240.5)	191.0 (141.8–243.8)	179.0 (139.0–240.0)	0.268
Creatinine, mg/dL	0.8 (0.6–1.1)	0.8 (0.7–1.0)	0.8 (0.6–1.1)	0.882
INR	1.1 (1.0–1.2)	1.1 (1.0–1.2)	1.1 (1.0–1.2)	0.213
PT, s	12.1 (11.2–13.2)	12.0 (11.1–13.2)	12.1 (11.3–13.2)	0.219
PTT, s	25.9 (23.9–28.3)	26.2 (24.4–29.0)	25.8 (23.8–28.1)	0.080
SpO2, %	85.0 (77.0–85.0)	84.0 (75.0–85.0)	85.0 (79.0–85.0)	< 0.001
Sodium, mEq/L	140.0 (137.0–142.0)	140.0 (137.0–142.0)	139.0 (137.0–142.0)	0.762
Potassium, mEq/L	3.2 (3.0–3.5)	3.1 (2.9–3.4)	3.3 (3.0–3.5)	< 0.001
Bicarbonate, mEq/L	19.0 (17.0–21.0)	19.0 (17.0–21.0)	19.0 (17.0–21.0)	0.336
GCS score	9.0 (6.0–13.0)	7.0 (4.0–10.0)	11.0 (7.0–14.0)	< 0.001
SAPS II score	34.0 (27.0–42.0)	35.0 (27.0–44.0)	34.0 (27.0–42.0)	0.633
APS III score	37.0 (28.0–52.0)	40.0 (29.0–54.0)	36.0 (27.0–51.0)	0.040
Charlson Comorbidity Index	4.0 (3.0–6.0)	5.0 (3.0–7.0)	4.0 (3.0–6.0)	0.020
ICU LOS, days	9.5 (3.8–17.0)	13.8 (8.3–21.8)	6.5 (2.9–13.8)	< 0.001
Hospital LOS, days	16.7 (8.7–25.1)	21.9 (15.3–33.9)	12.8 (5.9–21.6)	< 0.001
Male sex, *n* (%)	285 (56.4)	111 (58.7)	174 (55.1)	0.477
WFNS Grades IV–V, *n* (%)	350 (69.3)	155 (82.0)	195 (61.7)	< 0.001
Mechanical ventilation, *n* (%)	482 (95.4)	185 (97.9)	297 (94.0)	0.047
Endovascular intervention, *n* (%)	68 (13.5)	27 (14.3)	41 (13.0)	0.777
Propofol use, *n* (%)	289 (57.2)	108 (57.1)	181 (57.3)	1.000
Midazolam use, *n* (%)	235 (46.5)	98 (51.9)	137 (43.4)	0.078
Dexmedetomidine use, *n* (%)	239 (47.3)	108 (57.1)	131 (41.5)	< 0.001
Acetaminophen use, *n* (%)	263 (52.1)	86 (45.5)	177 (56.0)	0.028
Hyperlipidemia, *n* (%)	137 (27.1)	49 (25.9)	88 (27.8)	0.714
Diabetes, *n* (%)	109 (21.6)	37 (19.6)	72 (22.8)	0.462
CHD, *n* (%)	46 (9.1)	20 (10.6)	26 (8.2)	0.465
Cerebral ischemia, *n* (%)	27 (5.3)	12 (6.3)	15 (4.7)	0.569
COPD, *n* (%)	73 (14.5)	40 (21.2)	33 (10.4)	0.001
CHF, *n* (%)	60 (11.9)	25 (13.2)	35 (11.1)	0.561
Renal disease, *n* (%)	41 (8.1)	19 (10.1)	22 (7.0)	0.288
DVT, *n* (%)	29 (5.7)	16 (8.5)	13 (4.1)	0.066
UTI, *n* (%)	126 (25.0)	55 (29.1)	71 (22.5)	0.119

*Note*: Surgical approach was categorised as endovascular intervention (endovascular coil embolisation or flow‐diversion procedure; *n* = 68, 13.5%) versus “other” (comprising surgical clipping, external ventricular drain [EVD] placement, decompressive craniectomy, and other open neurosurgical procedures; *n *= 437, 86.5%). The two categories did not differ significantly between the POP and non‐POP groups (14.3% vs. 13.0%, *p* = 0.777). Because the MIMIC‐IV dataset does not provide a finer‐grained procedure code that distinguishes individual open‐surgery subtypes within the “other” category, the internal breakdown of the “other” group cannot be reported from this database. GCS (Glasgow Coma Scale) is presented as a descriptive continuous variable only. Due to high collinearity with WFNS grade (Pearson *r* = −0.79), GCS was excluded from all multivariable logistic regression models; WFNS grade was retained for its SAH‐specific clinical utility in risk stratification.

Abbreviations: APS III, Acute Physiology Score III; CCI, Charlson Comorbidity Index; CHD, coronary heart disease; CHF, congestive heart failure; COPD, chronic obstructive pulmonary disease; DBP, diastolic blood pressure; DVT, deep vein thrombosis; GCS, Glasgow Coma Scale; ICU, intensive care unit; INR, international normalized ratio; LOS, length of stay; POP, postoperative pneumonia; PT, prothrombin time; PTT, partial thromboplastin time; RDW, red blood cell distribution width; SAPS II, Simplified Acute Physiology Score II; SBP, systolic blood pressure; SpO_2_, pulse oxygen saturation; UTI, urinary tract infection; WBC, white blood cell count; WFNS, World Federation of Neurosurgical Societies.

Regarding disease severity, the POP group had significantly lower GCS scores (7.0 [4.0–10.0] vs. 11.0 [7.0–14.0], *p* < 0.001), a higher proportion of WFNS Grades IV–V (82.0% vs. 61.7%, *p* < 0.001), higher APS III scores (40.0 [29.0–54.0] vs. 36.0 [27.0–51.0], *p* = 0.040), and higher CCI (5.0 [3.0–7.0] vs. 4.0 [3.0–6.0], *p* = 0.020), indicating greater disease severity and comorbidity burden. SAPS II scores did not significantly differ between groups (*p* = 0.633).

Regarding vital signs and laboratory parameters, the POP group had higher heart rate (68.5 [59.0–79.0] vs. 65.0 [57.0–75.2] bpm, *p* = 0.024], lower SpO_2_ (84.0 [75.0–85.0] vs. 85.0 [79.0%–85.0%], *p* < 0.001], and lower potassium levels (3.1 [2.9–3.4] vs. 3.3 [3.0–3.5] mEq/L, *p* < 0.001). RDW showed a trend toward increase (*p* = 0.051), as did PTT (*p* = 0.080). Other laboratory parameters (hemoglobin, glucose, WBC, platelets, creatinine, INR, sodium, bicarbonate) did not significantly differ (all *p* > 0.05).

Regarding treatment, the POP group had higher mechanical ventilation utilization (97.9% vs. 94.0%, *p* = 0.047), significantly higher dexmedetomidine use (57.1% vs. 41.5%, *p* < 0.001), and lower acetaminophen use (45.5% vs. 56.0%, *p* = 0.028). Midazolam use showed an increasing trend (51.9% vs. 43.4%, *p* = 0.078). Propofol use (*p* = 1.000) and surgical approach (endovascular: 14.3% vs. 13.0%, *p* = 0.777) did not significantly differ. Surgical approach was categorized as endovascular intervention (comprising endovascular coil embolization and flow diversion) versus “other” open neurosurgical procedures.

Regarding comorbidities, the POP group had a significantly higher prevalence of COPD (21.2% vs. 10.4%, *p* = 0.001) and a trend toward increased DVT incidence (8.5% vs. 4.1%, *p* = 0.066). Other comorbidities (hyperlipidemia, diabetes, CHD, cerebral ischemia, CHF, renal disease) did not significantly differ. Additionally, pulmonary embolism (PE) was identified in 8 patients (1.6%) overall: 5 (2.6%) in the POP group and 3 (0.9%) in the non‐POP group (*p* = 0.271). Given the small number of PE cases and the absence of a significant between‐group difference, PE was not included as a separate covariate in the primary analyses.

### 3.2 Risk Factor Screening for POP

Univariable logistic regression results are presented in Table S1. Using *p* < 0.1 as the screening threshold, 14 variables were significantly associated or showed trend associations with POP occurrence: GCS score (OR [95% CI]: 0.849 [0.808–0.892], *p* < 0.001), WFNS Grades IV–V (OR [95% CI]: 2.829 [1.831–4.370], *p* < 0.001), potassium (OR [95% CI]: 0.451 [0.293–0.696], *p* < 0.001), dexmedetomidine use (OR [95% CI]: 1.883 [1.307–2.712], *p* = 0.001), COPD (OR [95% CI]: 2.302 [1.394–3.803], *p* = 0.001), acetaminophen use (OR [95% CI]: 0.656 [0.456–0.942], *p* = 0.022), CCI (OR [95% CI]: 1.068 [1.002–1.138], *p* = 0.042), DVT (OR [95% CI]: 2.156 [1.013–4.588], *p* = 0.046), APS III score (OR [95% CI]: 1.009 [1.000–1.017], *p* = 0.052), mechanical ventilation (OR [95% CI]: 2.959 [0.991–8.833], *p* = 0.052), midazolam use (OR [95% CI]: 1.407 [0.980–2.020], *p* = 0.064), heart rate (OR [95% CI]: 1.012 [0.999–1.025], *p* = 0.067), glucose (OR [95% CI]: 1.002 [1.000–1.005], *p* = 0.072), and UTI (OR [95% CI]: 1.416 [0.940–2.135], *p* = 0.096).

Based on the variable selection strategy, 12 variables were included in the final multivariable logistic regression model. Variable inclusion rationale: age and sex were forced as a priori confounders; WFNS grade was retained over GCS (greater SAH specificity; collinearity *r* = −0.79); APS III was excluded (highly correlated with SAPS II, *r* = 0.74, and both collinear with WFNS grade); DVT and UTI were excluded (postoperative complications, not risk factors, with reverse causation risk). All included variables had VIF < 2.1, indicating no serious multicollinearity.

Multivariable logistic regression results are shown in Figure 2A. Key findings included: (1) WFNS Grades IV–V was the factor most strongly associated with POP (OR [95% CI]: 2.373 [1.473–3.824], *p* < 0.001), suggesting that more severe neurological dysfunction increases POP risk, likely through impaired consciousness leading to weakened cough reflexes and reduced airway protection; (2) dexmedetomidine use was associated with approximately 2‐fold higher odds of POP (OR [95% CI]: 2.026 [1.330–3.085], *p* = 0.001), which may reflect confounding by indication, as dexmedetomidine is commonly used in more severely ill patients requiring prolonged sedation, though its potential immunomodulatory effects on pulmonary defense functions also warrant attention; (3) COPD was associated with 81.7% higher odds of POP (OR [95% CI]: 1.817 [1.029–3.208], *p* = 0.039), consistent with published literature; (4) Each 1 mEq/L increase in potassium was associated with a 47.4% lower odds of POP (OR [95% CI]: 0.526 [0.323–0.856], *p* = 0.010). The potassium association showed a graded, dose‐response pattern: POP occurred in 60 of 115 patients (52.2%) with admission potassium < 3.0 mEq/L, 94 of 251 (37.5%) with potassium 3.0–3.49 mEq/L, and 35 of 134 (26.1%) with potassium ≥ 3.5 mEq/L (*p* for trend < 0.001; per‐category OR = 0.569, 95% CI: 0.436–0.742). Relative to the <3.0 mEq/L reference category, the fully adjusted ORs were 0.573 (95% CI: 0.351–0.937, *p* = 0.026) for 3.0–3.49 mEq/L and 0.345 (95% CI: 0.190–0.624, *p* < 0.001) for ≥ 3.5 mEq/L, confirming that lower admission potassium was associated with greater POP risk; and (5) acetaminophen use was independently associated with lower POP odds (OR [95% CI]: 0.597 [0.399–0.892], *p* = 0.012), possibly related to its anti‐inflammatory and analgesic properties reducing pain‐induced restrictive respiratory movements, its antipyretic effect attenuating the neuro‐inflammatory cascade that predisposes to pulmonary infection, and inhibition of prostaglandin E2‐mediated immunosuppression.

**FIGURE 2:**
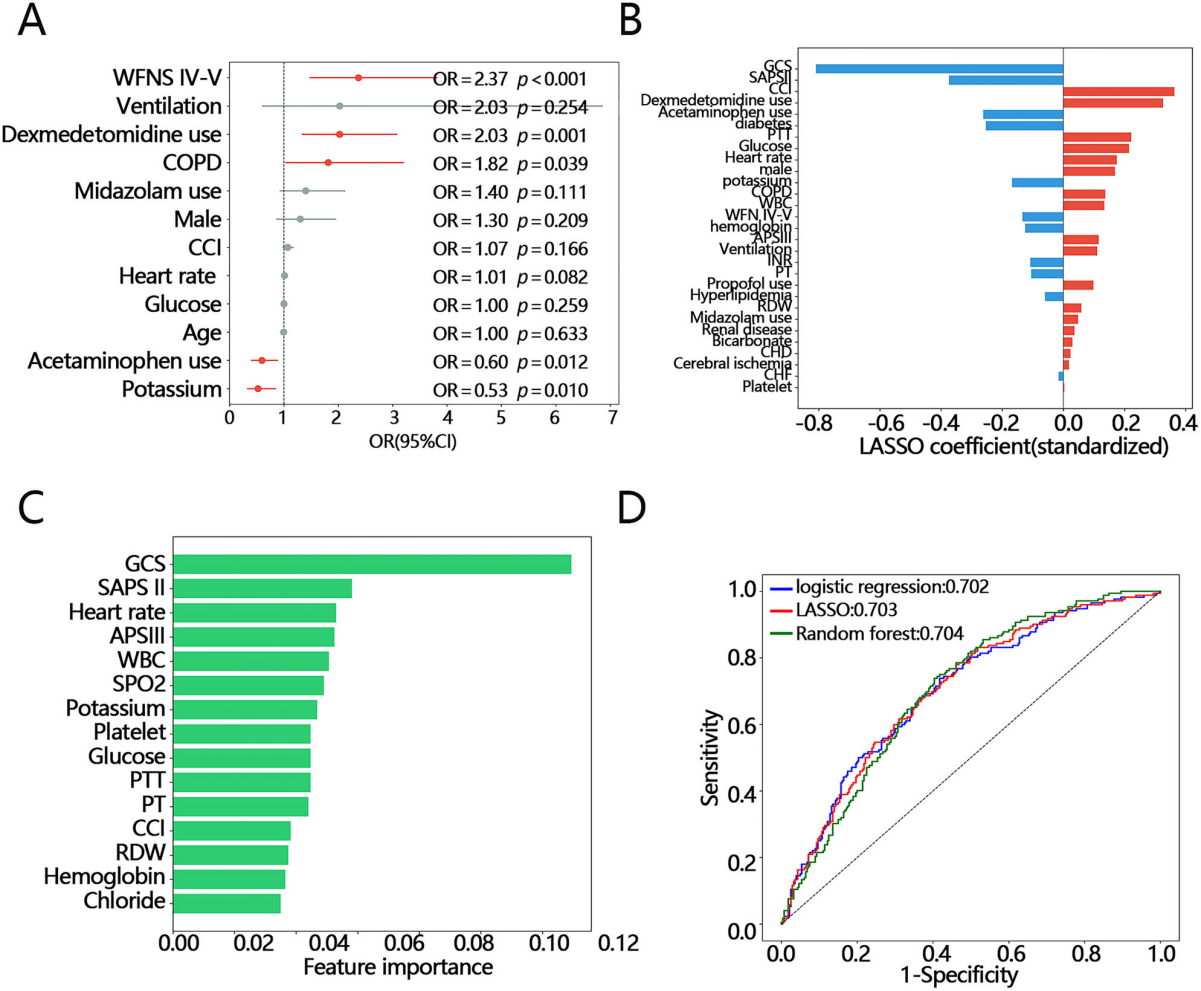
Risk factor screening for POP. (A) Forest plot of multivariable logistic regression; (B) variables selected by LASSO regression with standardized coefficients (red = positive/risk factor; blue = negative/protective factor); (C) top 15 variable importance from random forest model; (D) ROC curve comparison of three predictive models. APS III, Acute Physiology Score III; AUC, area under the curve; CCI, Charlson Comorbidity Index; CHD, coronary heart disease; CHF, congestive heart failure; CI, confidence interval; COPD, chronic obstructive pulmonary disease; DVT, deep vein thrombosis; UTI, urinary tract infection; GCS, Glasgow Coma Scale; INR, international normalized ratio; LASSO, least absolute shrinkage and selection operator; OR, odds ratio; POP, postoperative pneumonia; PT, prothrombin time; PTT, partial thromboplastin time; RDW, red blood cell distribution width; ROC, receiver operating characteristic; SAPS II, Simplified Acute Physiology Score II; SpO2, pulse oxygen saturation; WBC, white blood cell count; WFNS, World Federation of Neurosurgical Societies;

The overall model pseudo‐R‐squared was 0.106, AIC was 615.4, and BIC was 670.1. The Hosmer–Lemeshow goodness‐of‐fit test yielded chi‐squared = 16.77 (*p* = 0.033), indicating suboptimal model fit (*p* < 0.05 suggests the model may not adequately capture all relevant predictors); further model refinement may be warranted. Other included but nonsignificant variables were: age (OR [95% CI]: 0.996 [0.980–1.012], *p* = 0.633), sex (OR [95% CI]: 1.302 [0.863–1.964], *p* = 0.209), CCI (OR [95% CI]: 1.071 [0.972–1.181], *p* = 0.166), mechanical ventilation (OR [95% CI]: 2.028 [0.602–6.834], *p* = 0.254), midazolam use (OR [95% CI]: 1.403 [0.925–2.130], *p* = 0.111), heart rate (OR [95% CI]: 1.013 [0.998–1.027], *p* = 0.082), and glucose (OR [95% CI]: 1.002 [0.999–1.005], *p* = 0.259), with heart rate showing a borderline significant trend. It should be noted that the primary aim of the present multivariable model was to identify independent risk factors for POP rather than to construct an optimized clinical prediction tool; in this context, the AUC of 0.702 indicates acceptable discrimination. The calibration plot (Figure S1) showed adequate calibration overall, with mild overprediction in the highest decile of predicted probability, consistent with the borderline H–L result; this should be considered when interpreting model performance.

LASSO regression (10‐fold cross‐validation AUC = 0.703) and random forest analysis (10‐fold cross‐validation AUC = 0.704) further validated the logistic regression findings. In LASSO regression, among 35 candidate variables, 29 were retained (non‐zero coefficients) and 6 (age, surgery type, chloride, sodium, creatinine, SpO2) were shrunk to zero. The top 5 variables by absolute standardized coefficient were: GCS (−0.809), SAPS II (−0.374), CCI (+0.363), dexmedetomidine use (+0.326), and acetaminophen use (−0.262) (Figure 2B). In random forest, the top five variables by feature importance were: GCS (0.129), SAPS II (0.058), heart rate (0.053), APS III (0.053), and WBC (0.051); potassium ranked seventh (0.047) (Figure 2C).

Integrating results across three methods, the following variables were consistently identified as important predictors of POP: GCS/WFNS grade (neurological status), dexmedetomidine use, COPD, potassium level, acetaminophen use, CCI, and heart rate. The receiver operating characteristic (ROC) curves for all three models showed AUC values of 0.702–0.704, indicating moderate discriminatory ability (Figure 2D).

### 3.3 Association Between POP and Outcomes

Prognostic comparisons between the POP and non‐POP groups are shown in Figure 3. In‐hospital mortality was similar between groups (22.8% vs. 22.5%, *p* = 1.000), as were 30‐day mortality (5.8% vs. 2.8%, *p* = 0.155) and 90‐day mortality (9.0% vs. 6.3%, *p* = 0.349). However, the POP group showed a trend toward higher 1‐year mortality (14.3% vs. 8.5%, *p* = 0.061) (Figure 3A). Regarding LOS, the POP group had significantly longer ICU LOS (13.8 [8.3–21.8] vs. 6.5 [2.9–13.8] days, *p* < 0.001) and total hospital LOS (21.9 [15.3–33.9] vs. 12.8 [5.9–21.6] days, *p* < 0.001) (Figure 3B). Because in‐hospital deaths were recorded as a separate endpoint, the 30‐day, 90‐day, and 1‐year percentages above use the whole cohort as the denominator. Examined as three mutually exclusive tiers, in‐hospital death occurred in 43 of 189 POP patients (22.8%) and 71 of 316 non‐POP patients (22.5%) (Fisher's *p* >0.05), that is 114 of 505 patients (22.6%) overall. Among patients who survived to hospital discharge (POP, *n* = 146; non‐POP, *n* = 245), post‐discharge mortality was 11 of 146 (7.5%) versus 9 of 245 (3.7%) at 30 days (*p* = 0.102), 17 of 146 (11.6%) versus 20 of 245 (8.2%) at 90 days (*p* = 0.285), and 27 of 146 (18.5%) versus 27 of 245 (11.0%) at 1 year (*p* = 0.048). When in‐hospital death was combined with post‐discharge death within 1 year, cumulative mortality was 70 of 189 (37.0%) in the POP group versus 98 of 316 (31.0%) in the non‐POP group (Fisher's *p* = 0.173; unadjusted OR = 1.309, 95% CI: 0.895–1.912, *p* = 0.165).

**FIGURE 3:**
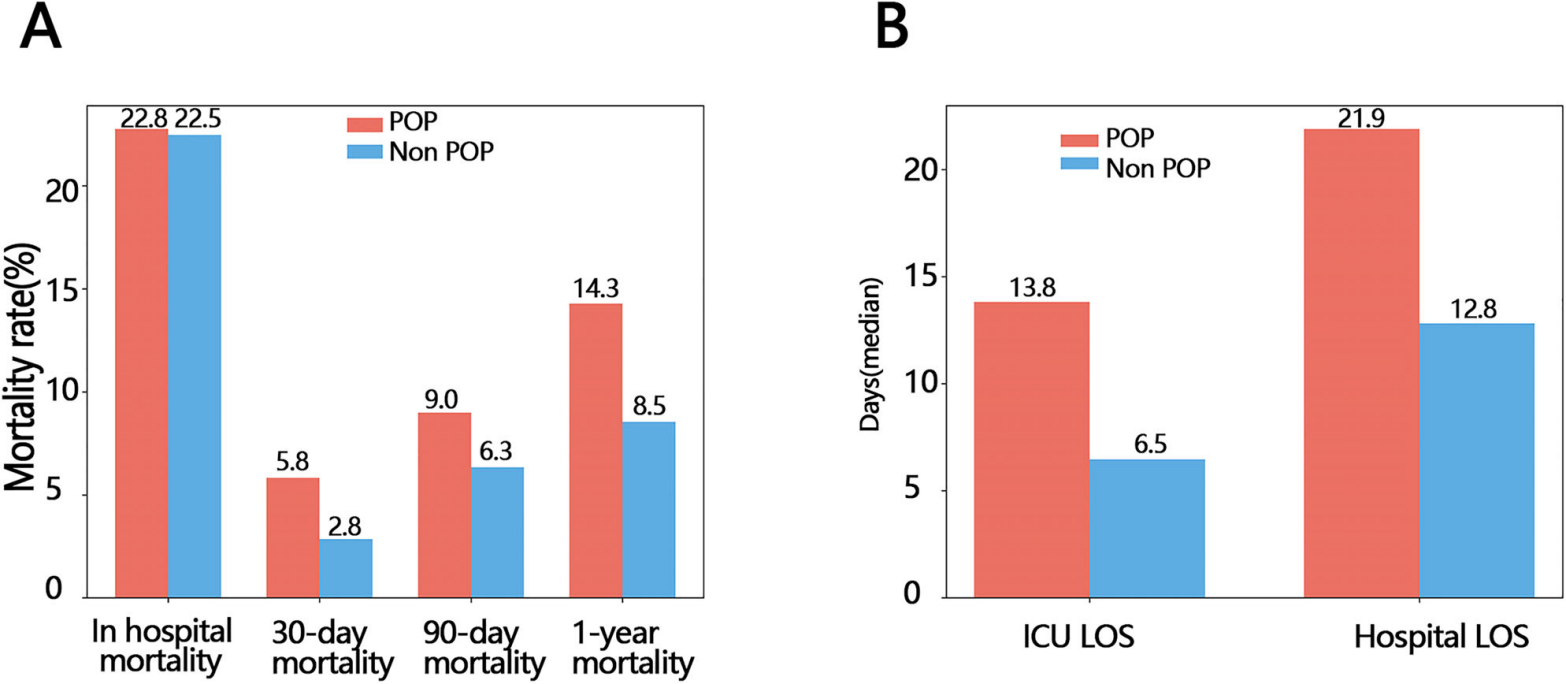
Prognostic comparison between the POP and the non‐POP groups. (A) Mortality at different timepoints; (B) median ICU LOS and total hospital LOS. ICU, intensive care unit; LOS, length of stay; POP, postoperative pneumonia.

Progressive adjustment analysis for the association between POP and mortality outcomes showed that across all four models, the OR for POP and in‐hospital mortality consistently approximated 1.0 without statistical significance (Model 1: OR [95% CI]: 1.016 [0.661–1.563], *p* = 0.941; Model 2: OR [95% CI]: 0.990 [0.636–1.543], *p* = 0.966; Model 3: OR [95% CI]: 1.054 [0.654–1.698], *p* = 0.831; Model 4: OR [95% CI]: 1.030 [0.628–1.688], *p* = 0.908). This indicates no independent association between POP and in‐hospital mortality after adjusting for disease severity and other confounders (Figure 4A). The 30‐day and 90‐day mortality were nonsignificant across all models, though OR values were consistently above 1, with limited sample size potentially contributing to the lack of statistical significance (Figure 4B,C). For 1‐year mortality (Figure 4D), POP was associated with increased risk in the unadjusted model (OR [95% CI]: 1.784 [1.012–3.145], *p* = 0.045), with borderline significance after adjusting for age and sex (OR [95% CI]: 1.780 [1.000–3.167], *p* = 0.050). However, after further adjusting for disease severity (WFNS grade, SAPS II), the association attenuated to nonsignificance (OR [95% CI]: 1.678 [0.931–3.023], *p* = 0.085), and the fully adjusted OR further decreased to 1.555 (OR [95% CI]: 1.555 [0.855–2.829], *p* = 0.148). This progressive attenuation pattern suggests that the univariable association between POP and long‐term mortality is likely driven by confounding from disease severity rather than an independent effect of pneumonia itself.

**FIGURE 4:**
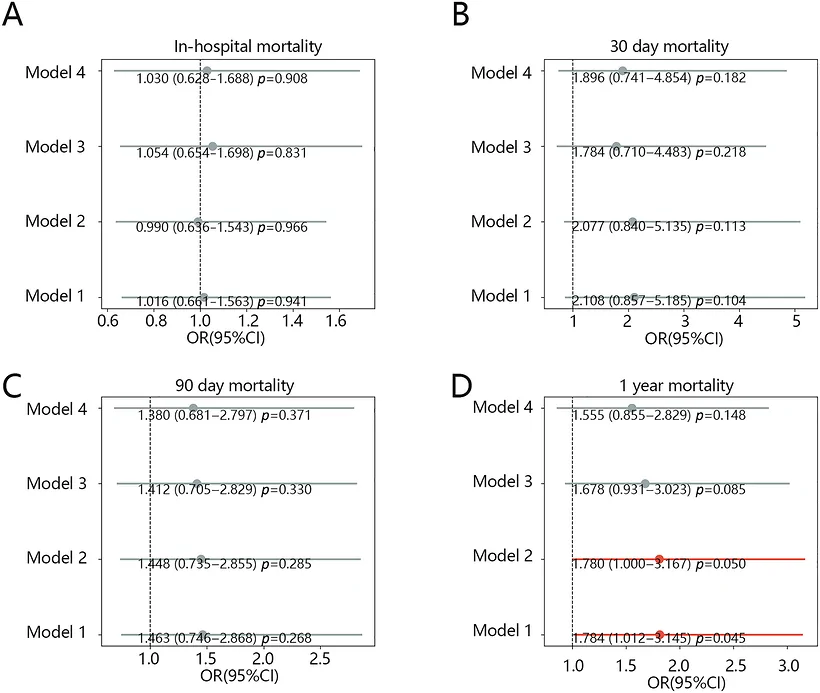
Forest plots of the progressively adjusted association between POP and mortality at different time points. (A) In‐hospital mortality; (B) 30‐day mortality; (C) 90‐day mortality; (D) 1‐year mortality across four progressively adjusted models. Mortality at 30 days, 90 days, and 1 year refers to death occurring after hospital discharge among patients who survived to discharge; in‐hospital deaths were analyzed separately. Model 1: without adjustment; Model 2: adjusting for age, sex; Model 3: adjusting for Age, Sex, WFNS, SAPSII; Model 4: adjusting for age, sex, WFNS, SAPSII, mechanical ventilation, CCI, diabetes, COPD. CCI, Charlson Comorbidity Index; CI, confidence interval; COPD, chronic obstructive pulmonary disease; OR, odds ratio; POP, postoperative pneumonia; SAPS II, Simplified Acute Physiology Score II; WFNS, World Federation of Neurosurgical Societies;

In stark contrast to the mortality results, the association of POP with LOS remained highly significant across all adjustment models (Figure 5A). For ICU LOS, the fully adjusted model showed that ICU LOS in the POP group was 1.651 times that of the non‐POP group (95% CI: 1.414–1.927, *p* < 0.001). For total hospital LOS, the POP group had 1.765 times the LOS of the non‐POP group (95% CI: 1.511–2.061, *p* < 0.001) (Figure 5B). These results indicate that while POP did not significantly increase mortality risk, it substantially prolonged ICU and hospital LOS, increasing healthcare resource consumption and economic burden.

**FIGURE 5:**
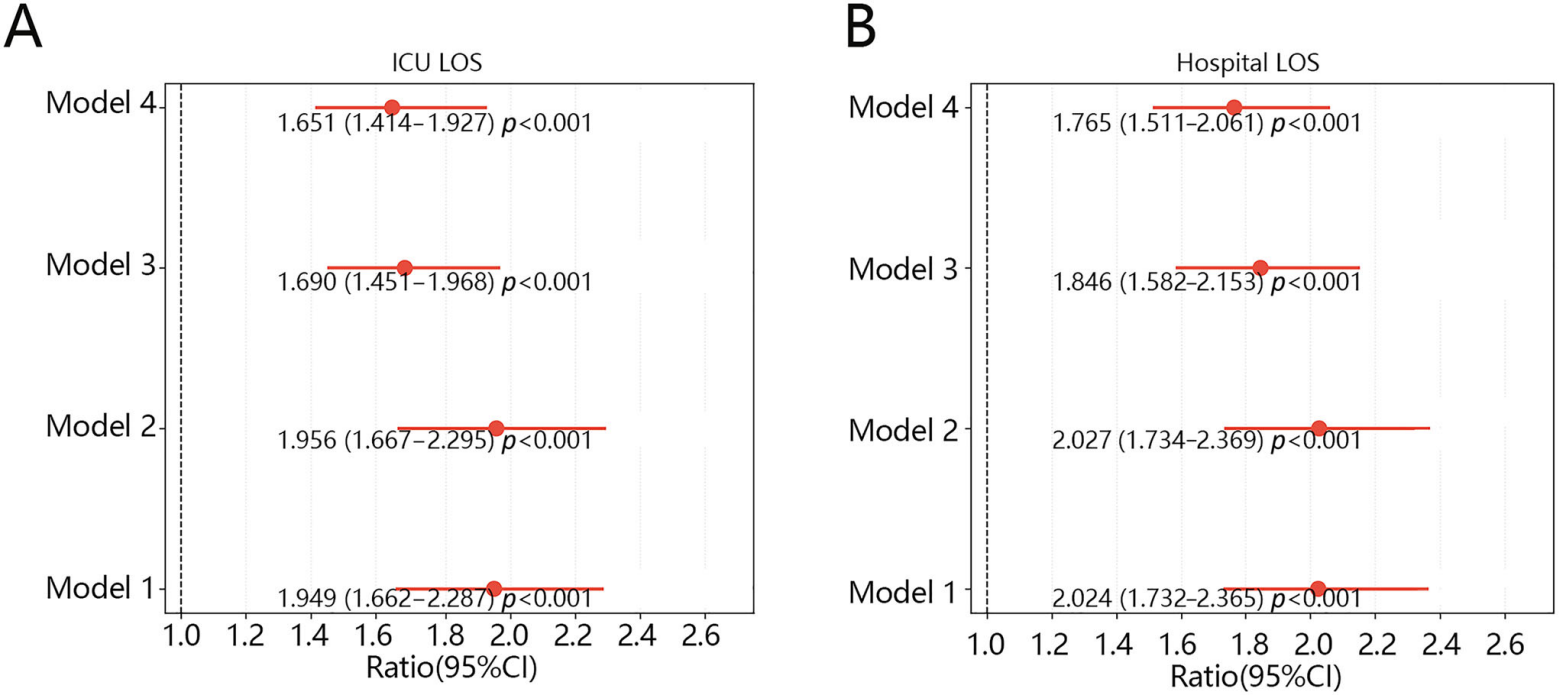
Progressive adjustment analysis of the association between POP and LOS. (A) ICU LOS; (B) total hospital LOS across four progressively adjusted models. Model 1: without adjustment; Model 2: adjusting for Age, Sex; Model 3: adjusting for Age, Sex, WFNS, SAPSII; Model 4: adjusting for Age, Sex, WFNS, SAPSII, mechanical ventilation, CCI, diabetes, COPD Abbreviations: CCI, Charlson Comorbidity Index; CI, confidence interval; COPD, chronic obstructive pulmonary disease; ICU, intensive care unit; LOS, length of stay; POP, postoperative pneumonia; SAPS II, Simplified Acute Physiology Score II; WFNS, World Federation of Neurosurgical Societies;

Because 95.4% of the cohort received mechanical ventilation, outcomes were additionally compared within ventilation strata (Table S2). Among ventilated patients (*n* = 482; POP, *n* = 185; non‐POP, *n* = 297), in‐hospital mortality was 41 of 185 (22.2%) versus 67 of 297 (22.6%) (*p* = 1.000), and post‐discharge mortality among the 144 POP and 230 non‐POP hospital survivors was 11 (7.6%) versus 9 (3.9%) at 30 days (*p* = 0.156), 17 (11.8%) versus 19 (8.3%) at 90 days (*p* = 0.282), and 27 (18.8%) versus 26 (11.3%) at 1 year (*p* = 0.049). POP remained associated with longer ICU LOS (13.9 [8.6–21.8] vs. 6.7 [3.0–14.2] days, *p* < 0.001) and total hospital LOS (22.1 [15.4–34.0] vs. 12.9 [6.0–21.8] days, *p* < 0.001), closely reproducing the whole‐cohort estimates.

In the non‐ventilated subgroup (*n* = 23; POP, *n* = 4; non‐POP, *n* = 19), in‐hospital mortality was 2 of 4 (50.0%) versus 4 of 19 (21.1%) (Fisher's *p* = 0.270), and among the 2 POP and 15 non‐POP hospital survivors, only a single post‐discharge death occurred (1 of 15 non‐POP patients at 90 days and 1 year; 0 of 2 POP patients), so no meaningful comparison of follow‐up mortality was possible. POP prevalence differed between ventilation strata (38.4% vs. 17.4%; Fisher's *p* = 0.047). The non‐ventilated subgroup was too small to support inferential testing or multivariable modeling, and the ventilation‐stratified estimates are therefore presented descriptively only.

### 3.4 Robustness Analysis of the POP‐LOS Association

#### 3.4.1 Subgroup Analysis

Subgroup analyses stratified by age, sex, WFNS grade, COPD, CCI, and dexmedetomidine use are presented in Table S3. POP significantly prolonged LOS in the vast majority of subgroups (*p* < 0.001) with highly consistent effect directions, indicating broad generalizability. Two statistically significant interactions were identified: (1) COPD as an effect modifier (total hospital LOS P interaction = 0.026): among non‐COPD patients, POP extended LOS by 2.177‐fold (*p* < 0.001), whereas among COPD patients, the effect was only 1.386‐fold (*p* = 0.071, nonsignificant). This may reflect that COPD patients already have longer baseline LOS, diluting the marginal incremental effect of POP; the small COPD subgroup (*n* = 73) may also limit statistical power. (2) Dexmedetomidine as an effect modifier (total hospital LOS *p* interaction = 0.023): the POP effect was stronger in patients not receiving dexmedetomidine (2.368‐fold vs.1.628‐fold), suggesting that POP exerts a more pronounced additional impact on LOS in relatively less severely ill patients not receiving dexmedetomidine. Additionally, for ICU LOS, age stratification showed a borderline significant interaction trend (*p* interaction = 0.065), with a larger POP effect in younger patients (< 60 years: 2.280‐fold vs.1.666‐fold).

#### 3.4.2 Multivariable Log‐Linear Regression Model for Hospital LOS

A comprehensive multivariable log‐linear regression model including 13 variables was constructed (Table S4). For total hospital LOS (*N* = 478, R‐squared = 0.339): POP (ratio = 1.541, *p* < 0.001), WFNS Grades IV–V (ratio = 1.207, *p* = 0.124), dexmedetomidine use (ratio = 1.351, *p* < 0.001), and CCI (ratio = 1.073, *p* < 0.001) were independently associated with prolonged LOS; higher admission potassium (ratio = 0.543, *p* < 0.001) was independently associated with shorter hospital LOS. The model explained 33.9% of the variance in hospital LOS.

In the sensitivity analysis restricted to in‐hospital survivors (*N* = 391), the independent associations of POP with total hospital LOS (ratio = 1.576, *p* < 0.001), ruling out the alternative explanation that “pneumonia patients had longer LOS solely because they were awaiting death” (Table S5).

### 3.5 Sensitivity Analyses for Time‐Related Bias

Considering that POP is a time‐varying event during hospitalization and the current data only provides binary coding, sensitivity analyses were conducted to evaluate the robustness of the main conclusions. In the IPTW/doubly robust analysis, after constructing propensity scores and applying stabilized inverse probability weighting, covariate balance substantially improved, with post‐weighting SMD values all falling to approximately 0.05 or below (Figure 6A). The weighted doubly robust model showed: POP was not significantly associated with in‐hospital mortality (OR = 0.962, 95% CI: 0.599–1.546, *p* = 0.874), but remained highly significantly associated with ICU LOS (ratio = 1.682, 95% CI: 1.446–1.957, *p* < 0.001) and total hospital LOS (ratio = 1.861, 95% CI: 1.611–2.151, *p* < 0.001) (data not shown).

**FIGURE 6:**
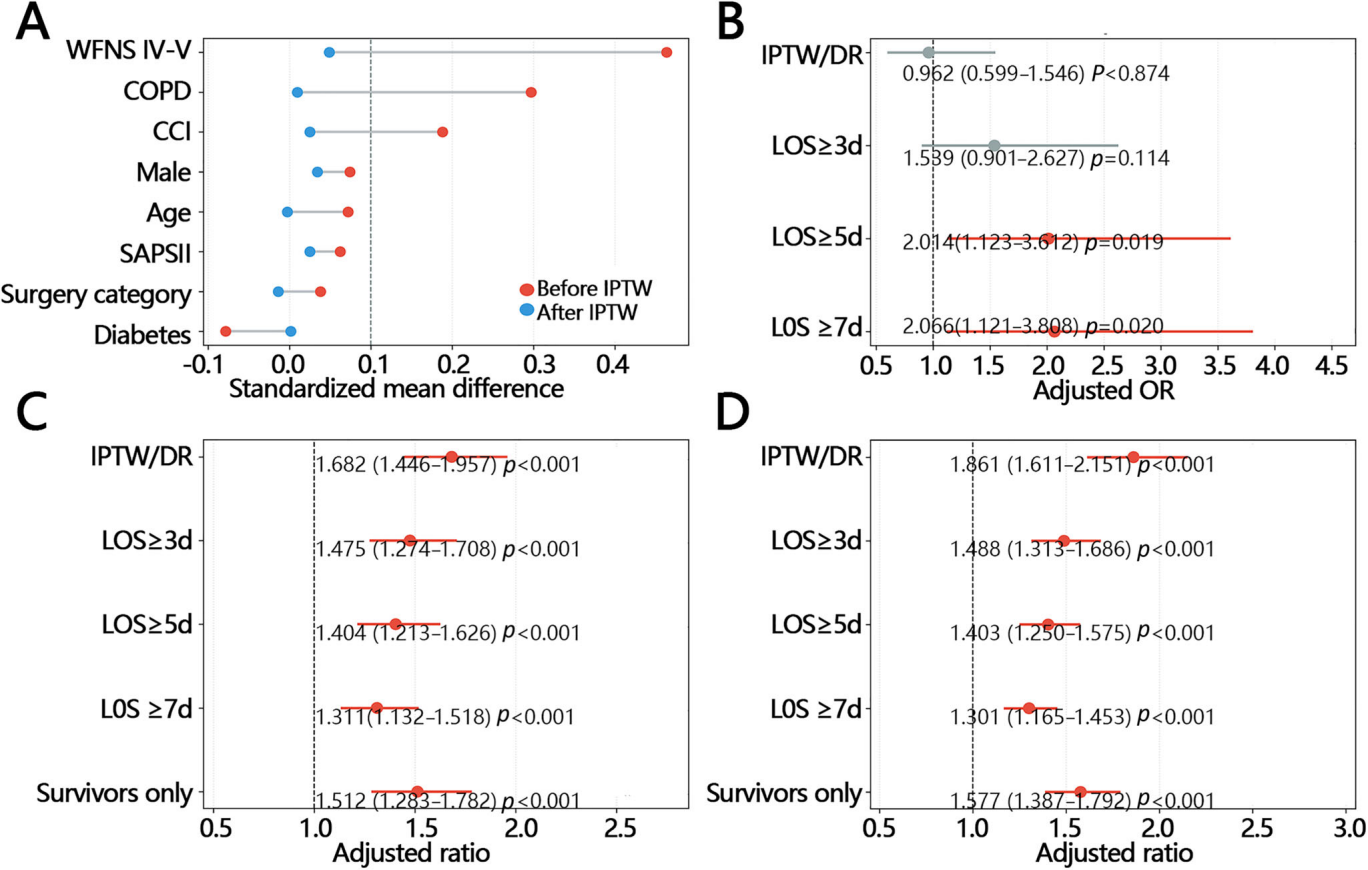
Supplementary sensitivity analyses for time‐related bias. (A) Covariate balance (SMD) before and after IPTW; (B) forest plot of in‐hospital mortality sensitivity analyses; (C) forest plot of ICU LOS sensitivity analyses; (D) forest plot of total hospital LOS sensitivity analyses. IPTW, inverse probability of treatment weighting; SMD, standardized mean difference; OR, odds ratio; CI, confidence interval; ICU, intensive care unit; LOS, length of stay.

Short‐stay exclusion and survivor‐restricted analyses confirmed highly stable directions and significance for LOS outcomes (Figure 6C,D). Regardless of restricting to total hospital LOS ≥ 3, ≥ 5, or ≥ 7 days, the POP effect on ICU and total hospital LOS remained significant (all *p* < 0.001); among in‐hospital survivors only, the association persisted (ICU LOS ratio = 1.512, 95% CI: 1.283–1.782; total hospital LOS ratio = 1.577, 95% CI: 1.387–1.792; both *p* < 0.001). In contrast, the in‐hospital mortality results were less stable across different restriction conditions (Figure 6B), suggesting that the mortality outcome is more susceptible to changes in analytical framework and sample composition and should be interpreted cautiously.

In the Fine–Gray subdistribution hazard model treating “in‐hospital death” and “alive discharge” as competing events, POP was not significantly associated with the subdistribution hazard of in‐hospital death (Figure 7C; sHR = 0.936, 95% CI: 0.631–1.390, *p* = 0.745), but was significantly associated with a reduced subdistribution hazard of alive discharge (sHR = 0.655, 95% CI: 0.526–0.815, *p* < 0.001). This suggests that POP primarily affects the discharge process and hospitalization burden rather than in‐hospital mortality risk itself.

**FIGURE 7:**
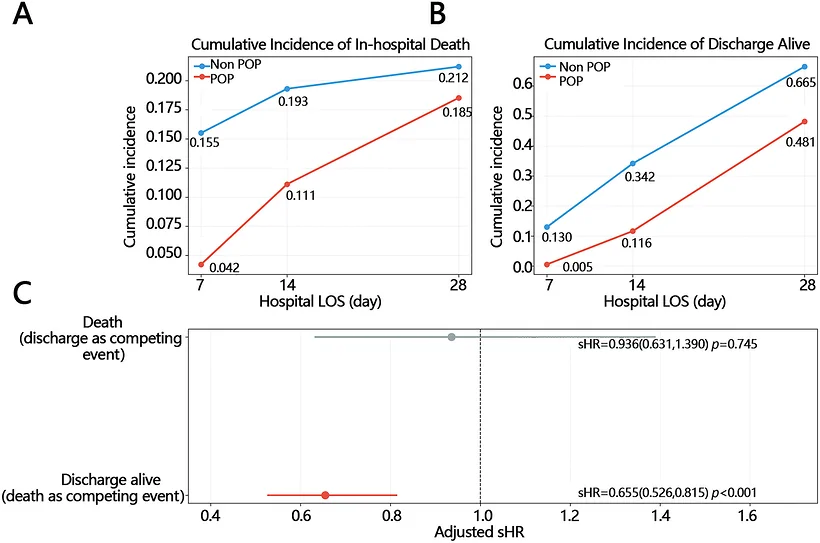
Competing risk analysis. (A) Cumulative incidence function for in‐hospital death; (B) cumulative incidence function for alive discharge; (C) Fine–Gray subdistribution hazard model results. Models uniformly adjusted for POP, age, sex, WFNS grade, SAPS II, mechanical ventilation, CCI, diabetes, and COPD. CIF, cumulative incidence function; COPD, chronic obstructive pulmonary disease; LOS, length of stay; POP, postoperative pneumonia; sHR, subdistribution hazard ratio; WFNS, World Federation of Neurosurgical Societies.

CIF further supported these findings. At Days 7, 14, and 28, the cumulative probability of alive discharge was consistently lower in the POP group (0.005, 0.116, and 0.481, respectively) compared with the non‐POP group (0.130, 0.342, and 0.665) (Figure 7B); the cumulative probability of in‐hospital death did not show a stable increase pattern, contradicting the main model conclusions (Figure 7A). Combining the main analysis and supplementary sensitivity analyses, POP primarily delays patients’ progress toward the “alive discharge” hospitalization endpoint.

### 3.6 Microbiological Characterization of Respiratory Isolates

In the cohort of 505 patients, 456 (90.3%) had at least one microbiology record and 299 (59.2%) had at least one respiratory specimen submitted (sputum, bronchoalveolar lavage, mini‐bronchoalveolar lavage, bronchial washings, or tracheal aspirate). A total of 1105 respiratory specimens were submitted, of which 574 (51.9%) yielded at least one organism, leaving 215 patients (42.6% of the cohort) with at least one positive respiratory culture. Specimen collection was closely tied to POP status: at least one respiratory specimen was submitted for 178 of 189 POP patients (94.2%) but for only 121 of 316 non‐POP patients (38.3%), and no respiratory specimen was sent at all for 11 POP patients (5.8%) versus 195 non‐POP patients (61.7%). Because sampling was driven by clinical suspicion rather than by a standardized surveillance protocol, pathogen frequencies are reported descriptively for the culture‐positive subgroup and are not compared between POP and non‐POP patients.

Among the 215 patients with a positive respiratory culture, the first respiratory isolate was *S. aureus* in 61 patients (28.4%), a yeast that was not further speciated in 45 (20.9%), an unidentified Gram‐negative rod in 19 (8.8%), *Haemophilus influenzae* in 17 (7.9%), *Enterobacter* spp. in 14 (6.5%), *Klebsiella pneumoniae* and *Escherichia coli* in 9 each (4.2% each), *Pseudomonas aeruginosa* in 8 (3.7%), *Serratia marcescens* and *Streptococcus pneumoniae* in 6 each (2.8% each), *Moraxella catarrhalis* in 4 (1.9%), and a heterogeneous group of remaining organisms in 17 (7.9%) (Table S6). *S. aureus* was therefore the most frequently implicated pathogen, accounting for 245 of the 865 respiratory isolates (28.3%) and for 48 of the 145 culture‐positive POP patients (33.1%).

In‐hospital mortality among the 215 culture‐positive patients was 53 of 215 (24.7%) overall and differed across pathogen groups: 9 of 61 (14.8%) when the first isolate was *S. aureus*, 15 of 45 (33.3%) for yeast, 8 of 19 (42.1%) for unidentified Gram‐negative rods, 5 of 14 (35.7%) for *Enterobacter spp*., 4 of 9 (44.4%) for *K. pneumoniae*, 4 of 9 (44.4%) for *E. coli*, 2 of 8 (25.0%) for *P. aeruginosa*, 3 of 6 (50.0%) for *S. marcescens*, 1 of 4 (25.0%) for *M. catarrhalis*, 1 of 17 (5.9%) for *H. influenzae*, 0 of 6 (0.0%) for *S. pneumoniae*, and 1 of 17 (5.9%) for the remaining organisms. Within the culture‐positive POP subgroup, 35 of 145 patients (24.1%) died in hospital. These figures are unadjusted and the pathogen groups were small, so they should not be read as pathogen‐specific attributable mortality; notably, patients whose first isolate was *S. aureus* had lower baseline severity than those with other organisms (WFNS Grades IV–V, 45/61 [73.8%] vs. 132/154 [85.7%], Fisher's *p* = 0.048; SAPS II, median 30.0 [IQR 26.0–40.0] vs. 36.0 [30.0–43.8], *p* = 0.023), which most plausibly explains the lower crude mortality in that group.

Susceptibility results were available for 370 of the 865 respiratory isolates (42.8%). Yeast isolates had no susceptibility testing, so resistance could be assessed only for bacterial isolates, principally *S. aureus*. Among the 61 patients whose first isolate was *S. aureus*, oxacillin susceptibility was reported for 56 (91.8%): 11 were methicillin‐resistant (MRSA) and 45 methicillin‐susceptible (MSSA). In‐hospital mortality was 3 of 11 (27.3%) in MRSA and 6 of 45 (13.3%) in MSSA (Fisher's *p* = 0.358), and POP occurred in 9 of 11 (81.8%) and 35 of 45 (77.8%), respectively. The MRSA subgroup is too small for a reliable comparison, and no conclusion about a resistance‐associated mortality difference can be drawn. Positive MRSA screening cultures (nares surveillance swabs) were excluded from all pathogen analyses.

Respiratory cultures were frequently polymicrobial: of the 574 positive respiratory specimens, 386 (67.2%) grew a single organism and 188 (32.8%) grew two or more. All pathogen figures above refer to the first isolate recovered during the index hospitalization; the extract available to us contains no surgery or ICU‐admission timestamps, so isolates cannot be restricted to those recovered after the operation and should be interpreted as respiratory isolates recovered during the index hospitalization rather than as strictly postoperative pathogens. Two further constraints apply. First, because cultures were ordered at clinical discretion, the absence of a positive culture cannot be equated with the absence of bacterial pneumonia, and the 44 POP patients without a positive respiratory culture (23.3% of the POP group) include both patients who were never sampled and patients whose cultures were negative. Second, non‐bacterial testing was sparse (fungal culture in 139 patients, respiratory viral PCR in 20, *Legionella* testing in 51, acid‐fast bacilli testing in 46, and *Pneumocystis* testing in 16), so non‐bacterial pathogens are almost certainly under‐ascertained. The distribution reported here therefore describes what was recovered under routine care rather than the full microbiological spectrum of POP in this population.

## 4 Discussion

This study systematically investigated the association between POP and prognosis in non‐traumatic SAH patients using the MIMIC‐IV database. The principal findings were: (1) the POP incidence was 37.4%, with high WFNS grade, dexmedetomidine use, and COPD as factors independently associated with increased POP odds, and higher admission potassium levels and acetaminophen use as factors independently associated with lower POP odds; (2) after multivariable adjustment, POP was not independently associated with in‐hospital mortality but was independently associated with significantly prolonged ICU and total hospital LOS; and (3) these conclusions were validated through IPTW/doubly robust analysis, short‐stay exclusion analysis, survivor‐restricted analysis, and Fine‐Gray competing risk models, demonstrating robust stability.

The POP incidence of 37.4% in this study exceeds the 10%–30% reported in previous literature (Jin et al. 2023; Li et al. 2024; Yuan et al. 2022). This difference may be attributable to our inclusion criteria: only patients receiving postoperative sedation were included, who typically have more severe illness and require longer mechanical ventilation support, thus conferring higher POP risk. Additionally, the high proportion of WFNS Grades IV–V patients (69.3%) in the MIMIC‐IV database may contribute to the elevated POP rate. It is important to acknowledge that within the category of non‐traumatic SAH, aneurysmal SAH (aSAH) carries the highest risk and most severe complications, accounting for approximately 80% of cases and being associated with the greatest morbidity and mortality. In our cohort, 14.3% of POP patients and 13.0% of non‐POP patients underwent endovascular intervention (a proxy for aneurysmal etiology), with no significant between‐group difference (*p* = 0.777). The relatively low proportion of endovascular cases in this sedation‐selected cohort likely reflects enrichment for the most severely ill patients requiring open neurosurgical approaches. A dedicated analysis restricted to aSAH would further refine the generalizability of our findings to the highest‐risk population.

WFNS high grade, as the factor most strongly associated with POP and consistently validated across all three analytical methods, aligns with previous findings: severe neurological dysfunction following SAH leads to reduced consciousness, weakened cough reflexes, and swallowing dysfunction, thereby increasing aspiration risk and promoting POP (Solenski et al. 1995; Wartenberg and Mayer 2006). Frontera et al. (2008) similarly reported that SAH patients with lower admission GCS scores had significantly increased nosocomial infection risk. Aspiration pneumonia deserves particular attention as a mechanistic pathway linking high WFNS grade to POP. Severely impaired consciousness (WFNS Grades IV–V) compromises laryngeal protective reflexes and swallowing coordination, markedly increasing the risk of oropharyngeal secretion and gastric content aspiration into the lower airways. In the MIMIC‐IV dataset, respiratory culture results are available for the specimens collected during the index hospitalization, but aspiration events are not documented, and the extract contains no surgery or ICU‐admission timestamps; therefore, we were unable to formally classify POP subtypes. Nevertheless, the strong association between WFNS Grades IV–V and POP in this cohort is consistent with aspiration being a major contributory mechanism (Frontera et al. 2008).

The microbiological profile of the culture‐positive patients provides context for this mechanistic account. *S. aureus* was the single most frequent respiratory isolate, recovered as the first isolate in 61 of 215 culture‐positive patients (28.4%), followed by yeast and Gram‐negative bacilli; this distribution is more consistent with aspiration‐ or healthcare‐associated pneumonia than with classical community‐acquired pneumococcal pneumonia, and it makes staphylococcal coverage a relevant consideration when empirical antimicrobial therapy is chosen for this population. The predominance of *S. aureus* also carries a resistance dimension: 11 of the 56 *S. aureus* isolates with oxacillin susceptibility reported were methicillin‐resistant, and crude in‐hospital mortality was numerically higher in those patients (27.3% vs. 13.3%), although the difference was not statistically significant and the subgroup was small. No pathogen‐specific attributable mortality can be inferred, both because the pathogen groups differed at baseline and because cultures were obtained at clinical discretion; the descriptive data nonetheless support the aspiration pathway outlined above and argue for prospective microbiological surveillance with standardized sampling in future studies of POP after SAH.

The association between dexmedetomidine use and approximately two‐fold increased POP risk warrants careful interpretation. In observational studies, this association may be subject to confounding by indication: dexmedetomidine is commonly administered to critically ill patients requiring prolonged continuous sedation, who inherently have higher baseline odds of POP (Barr et al. 2013; Shehabi et al. 2019). However, some evidence suggests that dexmedetomidine may influence host defense through its immunomodulatory properties (Meng et al. 2018). Specifically, dexmedetomidine acts on α2‐adrenergic receptors on immune cells, potentially suppressing natural killer cell activity and neutrophil oxidative burst, which may impair early pulmonary bacterial clearance. In addition, its prolonged sedative effect suppresses spontaneous airway maneuvers (coughing, deep breathing) that normally protect against secretion retention. Clinicians should therefore balance the well‐established benefits of dexmedetomidine for agitation control and delirium prevention (Reade and Finfer 2014) against its potential to elevate POP risk—particularly in patients with concomitant WFNS Grades IV–V or COPD, where the POP risk is already elevated. Prospective studies are needed to clarify the causal direction of this association.

COPD as an independent risk factor for POP is consistent with clinical experience and published literature. COPD patients have higher odds of postoperative pulmonary infection due to chronic airway inflammation, impaired mucociliary clearance, and respiratory muscle dysfunction (Kalil et al. 2016). The protective effect of potassium levels also has biological plausibility: hypokalemia can cause respiratory muscle weakness and impaired ventilatory function, while potentially affecting immune cell function and increasing infection susceptibility (Palmer and Clegg 2019). SAH patients frequently develop hypokalemia due to sympathetic activation and stress response, consistent with our finding of significantly lower potassium levels in the POP group. From a pathophysiological standpoint, potassium depletion impairs the Na^+^/K^+^‐ATPase‐dependent membrane polarization of alveolar macrophages and T lymphocytes, reducing their capacity for oxidative killing and cytokine secretion. Furthermore, catecholamine‐driven kaliuresis following the acute sympathetic surge of SAH may create a self‐reinforcing cycle in which hypokalemia, neurological depression, and pulmonary immune dysfunction converge to elevate POP risk (Palmer and Clegg 2019). Routine electrolyte monitoring with prompt potassium repletion may therefore constitute a pragmatic preventive strategy. Regarding acetaminophen's protective effect (OR = 0.597), beyond analgesia‐facilitated respiratory mechanics, its antipyretic action may attenuate the prostaglandin E2‐mediated immunosuppressive pathway, and its documented reduction of oxidative stress in critically ill patients (Graham et al. 2013) may preserve alveolar macrophage function. This finding merits prospective confirmation given its clinical actionability. In this study, COPD was defined based on pre‐existing ICD‐coded diagnoses. Pathophysiologically, pre‐existing COPD promotes POP through chronic airway colonization, impaired mucociliary clearance, and baseline immune dysregulation that is further exacerbated by the acute inflammatory milieu of SAH and the immunosuppressive effects of prolonged sedation. Regarding heart rate: although heart rate showed a borderline association with POP in univariable analysis and was retained in the multivariable model, it did not reach statistical significance after adjustment. Elevated admission heart rate may reflect the degree of sympathetic activation and hemodynamic stress following SAH, which are in turn associated with systemic immune dysregulation and vulnerability to pulmonary infection. It should be reported as a nonsignificant covariate for transparency rather than as a primary finding.

A central finding of this study is the absence of an independent association between POP and in‐hospital mortality. This result was highly consistent across unadjusted and progressively adjusted models (OR values all approximately 1.0), and was further confirmed by IPTW/doubly robust analysis and Fine–Gray competing risk analysis. While this finding may seem counterintuitive, as POP as a serious infectious complication would be expected to increase mortality risk, similar results have been reported in previous research. Wolkewitz et al. (2008), using competing risk analysis in ICU patients, found that nosocomial pneumonia significantly prolonged hospital LOS but did not directly increase mortality risk. Bekaert et al. (2011), in a large multicenter study, reported that the attributable mortality of ICU‐acquired pneumonia was substantially lower than traditional estimates. Possible explanations include: (1) pneumonia predominantly occurs in patients with already severe illness, whose mortality risk is primarily determined by the severity of the SAH; (2) under modern critical care conditions, POP, while prolonging treatment, can mostly be controlled through timely antimicrobial therapy, with limited marginal contribution to mortality; and (3) selection bias may exist, as patients surviving long enough to develop POP may inherently have a certain survival advantage. From a methodological perspective, competing risk theory provides an additional explanatory lens: POP occurs preferentially in patients with longer LOS, and longer LOS itself reflects a nonfatal but complex disease course. In this framework, POP functions primarily as a “discharge‐delaying” complication rather than a “death‐accelerating” event—a distinction that becomes apparent only when competing discharge events are explicitly modeled (Bekaert et al. 2011; Wolkewitz et al. 2008). This framing has important quality‐improvement implications: interventions targeting POP prevention may not substantially alter in‐hospital mortality rates, but carry significant potential to reduce ICU occupancy and healthcare resource consumption. It should be noted that for 1‐year mortality, an unadjusted trend was observed (*p* = 0.045) which attenuated to nonsignificance after adjustment (*p* = 0.148), and heart rate showed a borderline trend (*p* = 0.082); these results should be interpreted with caution given the limited sample size and potential residual confounding.

For 1‐year mortality, POP was associated with increased risk in the unadjusted model, but this association progressively attenuated to nonsignificance as disease severity variables were sequentially included. This attenuation pattern suggests that the apparent association between POP and long‐term mortality is likely explained by confounding from disease severity rather than an independent causal effect of POP. This endpoint was ascertained among patients who survived to hospital discharge; in‐hospital deaths were analyzed separately in the in‐hospital mortality models.

Notably, and in marked contrast to the mortality findings, the association of POP with LOS demonstrated high consistency and robustness across all analytical strategies. The fully adjusted model showed that total hospital LOS in the POP group was 1.765‐fold and ICU LOS was 1.651‐fold that of the non‐POP group, effects that remained stable in IPTW analysis, short‐stay exclusion analyses, and survivor‐restricted analyses. The Fine‐Gray competing risk analysis provided a more nuanced perspective on POP's impact on LOS. POP significantly reduced the subdistribution hazard of alive discharge, indicating that POP primarily delays patients’ progress toward the discharge endpoint. CIF further demonstrated that by Day 28, the cumulative probability of alive discharge in the non‐POP far exceeded that in the POP group, intuitively reflecting POP's obstructive effect on the discharge process.

Subgroup analyses revealed two clinically meaningful interactions. The effect modification by COPD suggests that COPD patients’ already prolonged baseline LOS dilutes the marginal incremental effect of POP. The effect modification by dexmedetomidine use (*p* interaction = 0.023) indicates that POP exerts a more pronounced additional impact on LOS in relatively less severely ill patients not receiving dexmedetomidine, possibly because these patients have shorter baseline LOS, making the relative prolongation from pneumonia more pronounced. These interaction findings have important clinical implications. Regarding the COPD interaction, the non‐significant POP effect on LOS within the COPD subgroup should not be interpreted as COPD patients being “protected” from POP‐related LOS prolongation; rather, their elevated baseline LOS and higher pneumonia background risk create a ceiling effect that compresses the detectable marginal contribution of individual POP episodes. Clinically, this underscores the importance of comprehensive pulmonary rehabilitation and preoperative optimization for COPD patients scheduled for SAH surgery. Regarding the dexmedetomidine interaction, the larger POP effect in patients not receiving dexmedetomidine suggests that in relatively less sedated, less critically ill patients, an episode of POP constitutes a qualitatively distinct insult that substantially restructures their hospitalization trajectory. This observation reinforces the argument that POP prevention efforts should be prioritized even in patients who appear less severely ill at admission, as the relative impact on their hospitalization course may be proportionally greater.

The significant prolongation of LOS by POP has important clinical implications. Extended hospitalization not only increases healthcare costs and resource consumption but may also expose patients to additional nosocomial complications, creating a vicious cycle (Diringer et al. 2011; Wartenberg et al. 2006). Therefore, active POP prevention is of substantial value for improving overall clinical management and health economic outcomes in SAH patients. Regarding immunocompromised comorbidities, the prevalence of diabetes mellitus (DM) in our cohort was 19.6% in the POP group versus 22.8% in the non‐POP group, and renal disease was present in 10.1% versus 7.0%, respectively. Neither DM nor renal disease reached statistical significance in univariable or multivariable analyses of POP risk. While DM‐associated immune dysfunction and uremia‐related immune suppression are recognized risk factors for nosocomial infection in general ICU populations, their independent contribution to POP in this specific SAH surgical cohort appeared to be overshadowed by the dominant effect of disease severity (WFNS grade) and treatment‐related factors.

Based on the findings of this study, the following clinical recommendations are proposed: (1) enhanced POP surveillance and preventive measures should be implemented for SAH patients with high WFNS grades and concomitant COPD, including oral care, early mobilization, and swallowing function assessment; (2) when using dexmedetomidine for postoperative sedation, clinicians should be aware of its potential association with higher POP odds and weigh sedation benefits against infection risk; (3) electrolyte management in SAH patients should be prioritized, with timely correction of hypokalemia; and (4) although POP did not significantly increase mortality risk, its substantial impact on LOS suggests it should be targeted as a key quality improvement metric.

This study has several limitations: (1) as a retrospective database study, unmeasured confounding cannot be completely excluded; (2) POP diagnosis was based on ICD codes, which may introduce coding bias, and specific pneumonia types (e.g., community‐acquired vs. ventilator‐associated) and severity information were unavailable; (3) the absence of precise POP onset timing precluded strict time‐varying exposure analyses; although multiple sensitivity analyses were conducted to evaluate time‐related bias, immortal time bias cannot be entirely eliminated; (4) the MIMIC‐IV database represents a single center, and external generalizability requires further validation; and (5) the relatively limited sample size (505 patients) may have affected statistical power for secondary outcomes such as 30‐day and 90‐day mortality. Post hoc power analysis indicated that with 505 patients and an observed POP prevalence of 37.4%, the study was adequately powered (> 80%) for the primary outcome (in‐hospital mortality) and the LOS outcomes, but was likely underpowered for detecting moderate‐sized effects on 30‐day and 90‐day mortality. Future multicenter studies with larger sample sizes are needed to provide more definitive estimates for these secondary mortality endpoints. (6) Functional outcomes (e.g., modified Rankin Scale) and neurological recovery data were not available in the current dataset; future work linking administrative and clinical registry data would allow a more comprehensive evaluation of POP's long‐term consequences on patient quality of life. The absence of mRS and GOS data is a significant limitation of MIMIC‐IV‐based research, as these scales are the standard endpoints for assessing neurological recovery in SAH trials and are essential for comprehensive prognostic evaluation. Future studies with access to timestamped POP onset data should employ landmark analysis or time‐varying Cox models to more rigorously address this bias. (7) Microbiological data, including pathogen identification, antibiotic sensitivity profiles, and drug‐resistant organism status, were available only for respiratory specimens obtained at clinical discretion rather than by a standardized surveillance protocol, so complete ascertainment cannot be assumed, and the pathogen groups were too small and too different in baseline severity to support pathogen‐specific attributable mortality estimates. POP subtypes (e.g., VAP vs. non‐VAP) could not be formally distinguished, and the extract available to us contained no surgery or ICU‐admission timestamps, so isolates could not be restricted to those recovered after the operation. Future studies with prospective microbiological surveillance are needed to address this gap. (8) Systematic neuromuscular impairment (NMI) assessments (e.g., MRC sum score) were not available in MIMIC‐IV; NMI is a recognized risk factor for aspiration and POP in critically ill patients, and its contribution to POP risk in this SAH cohort warrants prospective evaluation. (9) Additionally, the temporal relationship between medication exposure and POP onset cannot be confirmed from the available data: for dexmedetomidine, confounding by indication is likely, as it is preferentially administered to more severely ill patients requiring prolonged sedation; for acetaminophen, its use as an analgesic and antipyretic may reflect overall clinical management intensity rather than an independent causal protective effect on POP risk. (10) WFNS grade was not natively recorded in MIMIC‐IV and was derived from the admission GCS score combined with documented focal neurological deficits, as described in the Methods; this operationalization may have misclassified some patients, and the high proportion of WFNS Grades IV–V in our cohort (69.3%) alongside a comparatively low in‐hospital mortality (22.6%) suggests that severity may have been overestimated in some cases. (11) Because in‐hospital death and post‐discharge death were recorded as mutually exclusive endpoints, and follow‐up death indicators were populated only for patients discharged alive, in‐hospital and post‐discharge mortality could not be combined into a single time‐to‐event analysis; the follow‐up mortality models therefore code in‐hospital deaths as non‐events at follow‐up, which would tend to attenuate rather than inflate the estimated associations, and survivor‐restricted estimates are reported for comparison. (12) Only 23 patients (4.6%) did not receive mechanical ventilation, so the ventilation‐stratified comparisons are descriptive and statistically underpowered, and results for the nonventilated subgroup should not be interpreted inferentially.

## 5 Conclusions

Using the MIMIC‐IV database, this study described the epidemiological characteristics of POP and its association with prognosis in nontraumatic SAH patients who underwent surgery. POP was not significantly associated with in‐hospital mortality but was significantly correlated with longer ICU and total hospital LOS. High WFNS grade, dexmedetomidine use, and COPD were independently associated with higher odds of POP. These findings were consistent across multiple sensitivity analyses. Clinical practice should consider POP prevention and management, given its potential relevance to LOS and healthcare resource utilization.

## Author Contributions

**Hao Wang**: conceptualization, writing – original draft, data curation, funding acquisition, formal analysis. **Taotao Shi**: writing – original draft, methodology. **Tao Ge**: writing – original draft, formal analysis. **Hongcai Wang**: writing – original draft, investigation. **Maosong Chen**: writing – original draft, writing – review and editing, supervision, data curation.

## Funding

This work was supported by the Traditional Chinese Medicine Research Project of Zhejiang Province (grant no.: 2025ZL521).

## Ethics Statement

The Ethics Committee of The Affiliated Lihuili Hospital of Ningbo University deemed that this research is based on open‐source data, so the need for ethics approval was waived.

## Consent

The authors have nothing to report.

## Conflicts of Interest

The authors declare no conflicts of interest.

## Supporting information



Supplementary Figure 1. Calibration plot for the primary multivariable logistic regression model predicting POP. The x‐axis shows the mean predicted probability per decile and the y‐axis shows the observed proportion of POP events. The Hosmer‐Lemeshow goodness‐of‐fit test yielded Chi‐squared = 16.77 (P = 0.033), indicating modest miscalibration in some deciles, predominantly at higher predicted probabilities. The overall discriminative ability of the model (AUC = 0.702) remained acceptable for risk‐factor identification purposes. POP, postoperative pneumonia; AUC, area under the curve.

Supplementary Table 1. Univariate Logistic Regression Analysis of Risk Factors for Postoperative Pneumonia

Supplementary Table 2. Ventilation‐Stratified Comparison of POP, Mortality, and Length of Stay

Supplementary Table 3 Subgroup Analysis of POP and Hospital LOS (or ICU LOS)

Supplementary Table 4. Multivariable Log‐Linear Regression Model of Hospital LOS

Supplementary Table 5. Sensitivity Analysis

Supplementary Table 6. Respiratory Pathogens in Patients with a Positive Respiratory Culture

## Data Availability

The data that support the findings of this study are available from the corresponding author upon reasonable request.
