# Supplementary material for: Association Between Postoperative Pneumonia and Prognosis in Patients With Non‐Traumatic Subarachnoid Hemorrhage: An Analysis of the MIMIC‐IV Database
Source: Brain Behav. 2026 Nov 9;16(11):e71916. doi: 10.1002/brb3.71916 (PMC13746991; doi:10.1002/brb3.71916)
Supplement: Supplementary file 1 [file BRB3-16-e71916-s001.docx]

| **Supplementary Table 1. Univariate Logistic Regression Analysis of Risk Factors for Postoperative Pneumonia** | | |
| --- | --- | --- |
| Variable | OR(95%CI) | P |
| GCS | 0.849(0.808-0.892) | <0.001 |
| WFNS IV-V | 2.829(1.831-4.370) | <0.001 |
| Potassium | 0.451(0.293-0.696) | <0.001 |
| Dexmedetomidine use | 1.883(1.307-2.712) | <0.001 |
| COPD | 2.302(1.394-3.803) | 0.001 |
| Acetaminophen use | 0.656(0.456-0.942) | 0.022 |
| CCI | 1.068(1.002-1.138) | 0.042 |
| DVT | 2.156(1.013-4.588) | 0.046 |
| APSIII | 1.009(1.000-1.017) | 0.052 |
| Ventilation | 2.959(0.991-8.833) | 0.052 |
| Midazolam use | 1.407(0.980-2.020) | 0.064 |
| Heart rate | 1.012(0.999-1.025) | 0.067 |
| Glucose | 1.002(1.000-1.005) | 0.072 |
| UTI | 1.416(0.940-2.135) | 0.096 |
| RDW | 1.083(0.981-1.195) | 0.114 |
| Platelet | 1.002(0.999-1.004) | 0.138 |
| Renal disease | 1.494(0.786-2.839) | 0.221 |
| PT | 0.960(0.895-1.029) | 0.253 |
| INR | 0.645(0.304-1.373) | 0.255 |
| SpO2 | 0.995(0.987-1.004) | 0.270 |
| PTT | 1.016(0.987-1.045) | 0.274 |
| WBC | 1.018(0.985-1.051) | 0.288 |
| Hemoglobin | 0.959(0.881-1.044) | 0.332 |
| Chloride | 0.985(0.953-1.018) | 0.363 |
| CHD | 1.320(0.715-2.437) | 0.375 |
| Diabetes | 0.825(0.529-1.288) | 0.397 |
| Male | 1.161(0.806-1.672) | 0.421 |
| Cerebral ischemia | 1.36(0.623-2.972) | 0.440 |
| Age | 1.004(0.994-1.015) | 0.440 |
| CHF | 1.224(0.707-2.117) | 0.470 |
| SAPSII | 1.005(0.991-1.019) | 0.493 |
| Bicarbonate | 0.984(0.937-1.034) | 0.522 |
| Sodium | 0.990(0.954-1.029) | 0.621 |
| Hyperlipidemia | 0.907(0.603-1.363) | 0.638 |
| Surgery category | 1.118(0.663-1.886) | 0.676 |
| Propofol use | 0.994(0.691-1.431) | 0.976 |
| Creatinine | 1.000(0.778-1.285) | 1.000 |

Abbreviations: POP, postoperative pneumonia; RDW, red blood cell distribution width; WBC, white blood cell count; INR, international normalized ratio; PT, prothrombin time; PTT, partial thromboplastin time; SpO2, pulse oxygen saturation; GCS, Glasgow Coma Scale; SAPS II, Simplified Acute Physiology Score II; APS III, Acute Physiology Score III; CCI, Charlson Comorbidity Index; WFNS, World Federation of Neurosurgical Societies; COPD, chronic obstructive pulmonary disease; DVT, deep vein thrombosis; UTI, urinary tract infection; OR, odds ratio; CI, confidence interval; CHD, coronary heart disease; CHF, congestive heart failure.
