# Supplementary material for: Association Between Postoperative Pneumonia and Prognosis in Patients With Non‐Traumatic Subarachnoid Hemorrhage: An Analysis of the MIMIC‐IV Database
Source: Brain Behav. 2026 Nov 9;16(11):e71916. doi: 10.1002/brb3.71916 (PMC13746991; doi:10.1002/brb3.71916)
Supplement: Supplementary file 2 [file BRB3-16-e71916-s002.docx]

| **Supplementary Table 4. Multivariable Log-Linear Regression Model of Hospital LOS** | | |
| --- | --- | --- |
| Variable | Ratio (95%CI) | P |
| Potassium | 0.543(0.46-0.638) | <0.001 |
| COPD | 0.901(0.739-1.099) | 0.302 |
| GCS | 0.977(0.949-1.006) | 0.112 |
| SAPSII | 0.985(0.979-0.991) | <0.001 |
| Age | 0.996(0.991-1.001) | 0.149 |
| Heart rate | 1.005(1.000-1.010) | 0.044 |
| CCI | 1.073(1.037-1.111) | <0.001 |
| Acetaminophen use | 1.112(0.971-1.273) | 0.125 |
| Ventilation | 1.153(0.829-1.605) | 0.397 |
| Male | 1.200(1.044-1.379) | 0.010 |
| WFNS IV-V | 1.207(0.95-1.535) | 0.124 |
| Dexmedetomidine use | 1.351(1.176-1.552) | <0.001 |
| POP | 1.541(1.328-1.789) | <0.001 |

Abbreviations: LOS, length of stay; CI, confidence interval; COPD, chronic obstructive pulmonary disease; GCS, Glasgow Coma Scale; SAPS II, Simplified Acute Physiology Score II; CCI, Charlson Comorbidity Index; WFNS, World Federation of Neurosurgical Societies; POP, postoperative pneumonia.
