# Supplementary material for: Association Between Postoperative Pneumonia and Prognosis in Patients With Non‐Traumatic Subarachnoid Hemorrhage: An Analysis of the MIMIC‐IV Database
Source: Brain Behav. 2026 Nov 9;16(11):e71916. doi: 10.1002/brb3.71916 (PMC13746991; doi:10.1002/brb3.71916)
Supplement: Supplementary file 3 [file BRB3-16-e71916-s003.docx]

| **Supplementary Table 5. Sensitivity Analysis** | |  |
| --- | --- | --- |
| Outcome | Ratio (95%CI) | P |
| Hospital LOS | 1.576（1.370-1.812） | <0.001 |

Abbreviations: LOS, length of stay; CI, confidence interval.
