# Supplementary material for: Association Between Postoperative Pneumonia and Prognosis in Patients With Non‐Traumatic Subarachnoid Hemorrhage: An Analysis of the MIMIC‐IV Database
Source: Brain Behav. 2026 Nov 9;16(11):e71916. doi: 10.1002/brb3.71916 (PMC13746991; doi:10.1002/brb3.71916)
Supplement: Supplementary file 4 [file BRB3-16-e71916-s004.docx]

| **Supplementary Table 2. Ventilation-Stratified Comparison of POP, Mortality, and Length of Stay** | | | | | | | |
| --- | --- | --- | --- | --- | --- | --- | --- |
| Subgroups | N | In-hospital death | Post-discharge death among hospital survivors | | | ICU LOS | Hospital LOS |
|  |  | n (%) | 30-day, n (%) | 90-day, n (%) | 1-year, n (%) | median (IQR), days | median (IQR), days |
| Mechanical ventilation | 482 |  |  |  |  |  |  |
| POP | 185 | 41 (22.2) | 11 (7.6) | 17 (11.8) | 27 (18.8) | 13.9 (8.6-21.8) | 22.1 (15.4-34.0) |
| Non-POP | 297 | 67 (22.6) | 9 (3.9) | 19 (8.3) | 26 (11.3) | 6.7 (3.0-14.2) | 12.9 (6.0-21.8) |
| OR (95% CI) |  | 0.977 (0.629-1.519) | 2.031 (0.820-5.029) | 1.487 (0.745-2.965) | 1.811 (1.009-3.249) | — | — |
| P |  | 1.000 | 0.156 | 0.282 | 0.049 | <0.001 | <0.001 |
| No mechanical ventilation | 23 |  |  |  |  |  |  |
| POP | 4 | 2 (50.0) | 0 (0.0) | 0 (0.0) | 0 (0.0) | 3.3 (2.4-5.0) | 13.2 (7.4-19.6) |
| Non-POP | 19 | 4 (21.1) | 0 (0.0) | 1 (6.7) | 1 (6.7) | 5.3 (2.3-8.0) | 9.9 (4.7-17.8) |
| OR (95% CI) |  | 3.750 (0.396-35.544) | NE | NE | NE | — | — |
| P |  | 0.270 | 1.000 | 1.000 | 1.000 | 0.725 | 0.725 |

POP, postoperative pneumonia; ICU, intensive care unit; LOS, length of stay; OR, odds ratio; CI, confidence interval; IQR, interquartile range; NE, not estimable (no event in the POP group).

In-hospital death and post-discharge death were recorded as mutually exclusive endpoints. In-hospital mortality uses the full stratum as the denominator; the 30-day, 90-day, and 1-year post-discharge figures were ascertained only among patients who survived to hospital discharge. Mortality comparisons used the two-sided Fisher exact test; LOS comparisons used the Mann-Whitney U test. ORs for post-discharge death are unadjusted. POP prevalence differed between the ventilation strata (38.4% vs. 17.4%, Fisher P = 0.047). Because only 23 patients (4.6%) did not receive mechanical ventilation, that stratum is presented descriptively and should not be interpreted inferentially; no multivariable model was fitted within either stratum.
