# Supplementary material for: Association Between Postoperative Pneumonia and Prognosis in Patients With Non‐Traumatic Subarachnoid Hemorrhage: An Analysis of the MIMIC‐IV Database
Source: Brain Behav. 2026 Nov 9;16(11):e71916. doi: 10.1002/brb3.71916 (PMC13746991; doi:10.1002/brb3.71916)
Supplement: Supplementary file 5 [file BRB3-16-e71916-s005.docx]

| **Supplementary Table 3 Subgroup Analysis of POP and Hospital LOS (or ICU LOS)** | | | | | | | |
| --- | --- | --- | --- | --- | --- | --- | --- |
| Subgroups | N | Hospital LOS | | | ICU LOS | | |
|  |  | Ratio(95%CI) | P | Interaction P | Ratio(95%CI) | P | Interaction P |
| Age |  |  |  | 0.882 |  |  | 0.065 |
| Age < 60 | 244 | 2.021 (1.587-2.572) | <0.001 |  | 2.280 (1.805-2.879) | <0.001 |  |
| Age≥60 | 261 | 1.927 (1.580-2.351) | <0.001 |  | 1.666 (1.343-2.067) | <0.001 |  |
| Sex |  |  |  | 0.912 |  |  | 0.709 |
| Female | 220 | 2.014 (1.587-2.557) | <0.001 |  | 1.876 (1.456-2.416) | <0.001 |  |
| Male | 285 | 2.055 (1.672-2.526) | <0.001 |  | 2.019 (1.642-2.483) | <0.001 |  |
| WFNS Grade | |  |  | 0.073 |  |  | 0.629 |
| I-III | 155 | 2.334 (1.621-3.361) | <0.001 |  | 1.761 (1.261-2.459) | 0.001 |  |
| IV-V | 350 | 1.706 (1.445-2.014) | <0.001 |  | 1.652 (1.391-1.962) | <0.001 |  |
| COPD |  |  |  | 0.026 |  |  | 0.052 |
| No COPD | 432 | 2.177 (1.830-2.589) | <0.001 |  | 2.088 (1.752-2.490) | <0.001 |  |
| COPD | 73 | 1.386 (0.972-1.977) | 0.071 |  | 1.402 (0.929-2.115) | 0.106 |  |
| CCI |  |  |  | 0.743 |  |  | 0.788 |
| CCI < 4 | 184 | 1.915 (1.469-2.496) | <0.001 |  | 2.015 (1.534-2.646) | <0.001 |  |
| CCI≥4 | 321 | 2.034 (1.679-2.464) | <0.001 |  | 1.923 (1.574-2.349) | <0.001 |  |
| Dexmedetomidine | |  |  | 0.023 |  |  | 0.425 |
| No Dexmedetomidine | 266 | 2.368 (1.868-3.002) | <0.001 |  | 2.016 (1.588-2.559) | <0.001 |  |
| Dexmedetomidine used | 239 | 1.628 (1.348-1.966) | <0.001 |  | 1.767 (1.423-2.194) | <0.001 |  |

Abbreviations: POP, postoperative pneumonia; SAPS II, Simplified Acute Physiology Score II; CCI, Charlson Comorbidity Index; WFNS, World Federation of Neurosurgical Societies; COPD, chronic obstructive pulmonary disease; CI, confidence interval.
