# Supplementary material for: Association Between Postoperative Pneumonia and Prognosis in Patients With Non‐Traumatic Subarachnoid Hemorrhage: An Analysis of the MIMIC‐IV Database
Source: Brain Behav. 2026 Nov 9;16(11):e71916. doi: 10.1002/brb3.71916 (PMC13746991; doi:10.1002/brb3.71916)
Supplement: Supplementary file 6 [file BRB3-16-e71916-s007.docx]

| **Supplementary Table 6. Respiratory Pathogens in Patients with a Positive Respiratory Culture** | | | | | | | |
| --- | --- | --- | --- | --- | --- | --- | --- |
| Pathogen | | Patients | | In-hospital death | POP | Post-discharge death by 1 year† | Hospital LOS |
| Organism | Isolates, n | n | % (of 215) | n (%) | n (%) | n (%) | median (IQR), days |
| Staphylococcus aureus | 245 | 61 | 28.4 | 9 (14.8) | 48 (78.7) | 9/52 (17.3) | 21.8 (15.4-38.7) |
| Yeast, not further speciated | 186 | 45 | 20.9 | 15 (33.3) | 23 (51.1) | 5/30 (16.7) | 24.8 (17.4-32.4) |
| Unidentified Gram-negative rod | 69 | 19 | 8.8 | 8 (42.1) | 9 (47.4) | 3/11 (27.3) | 14.0 (8.0-21.5) |
| Haemophilus influenzae | 40 | 17 | 7.9 | 1 (5.9) | 11 (64.7) | 1/16 (6.2) | 23.7 (15.8-27.3) |
| Enterobacter spp. | 52 | 14 | 6.5 | 5 (35.7) | 8 (57.1) | 0/9 (0.0) | 23.6 (13.2-32.5) |
| Klebsiella pneumoniae | 47 | 9 | 4.2 | 4 (44.4) | 7 (77.8) | 3/5 (60.0) | 17.1 (12.2-22.1) |
| Escherichia coli | 25 | 9 | 4.2 | 4 (44.4) | 7 (77.8) | 1/5 (20.0) | 23.6 (19.7-24.5) |
| Pseudomonas aeruginosa | 45 | 8 | 3.7 | 2 (25.0) | 8 (100.0) | 2/6 (33.3) | 21.8 (19.7-27.2) |
| Serratia marcescens | 37 | 6 | 2.8 | 3 (50.0) | 4 (66.7) | 0/3 (0.0) | 10.6 (10.5-15.4) |
| Streptococcus pneumoniae | 15 | 6 | 2.8 | 0 (0.0) | 4 (66.7) | 0/6 (0.0) | 32.1 (22.7-44.6) |
| Moraxella catarrhalis | 12 | 4 | 1.9 | 1 (25.0) | 4 (100.0) | 2/3 (66.7) | 28.6 (22.5-40.2) |
| Other | 92 | 17 | 7.9 | 1 (5.9) | 12 (70.6) | 2/16 (12.5) | 23.0 (21.0-35.1) |
| Total | 865 | 215 | 100.0 | 53 (24.7) | 145 (67.4) | 28/162 (17.3) | 22.1 (14.8-33.5) |

POP, postoperative pneumonia; LOS, length of stay; IQR, interquartile range. Patients were classified by the first respiratory isolate recovered during the index hospitalization, so percentages of patients in a column sum to 100.0%; the isolate counts describe all 865 respiratory isolates and overlap within patients.

† Denominator is the number of patients in that row who survived to hospital discharge; in-hospital and post-discharge deaths were recorded as mutually exclusive endpoints.

Respiratory specimens comprised sputum, bronchoalveolar lavage, mini-bronchoalveolar lavage, bronchial washings, and tracheal aspirate. Screening specimens such as nasal MRSA surveillance swabs and entries flagged as cancelled, negative, or non-specific were excluded. Isolates were deduplicated on specimen identifier, organism name, and isolate number.

“Other” comprises organisms each accounting for fewer than 1.5% of the 215 patients, namely Stenotrophomonas maltophilia (n = 2), Klebsiella oxytoca (n = 2), beta-haemolytic streptococci not group A (n = 2), beta-haemolytic streptococcus group B (n = 1), Acinetobacter baumannii complex (n = 2), Citrobacter freundii complex (n = 1), Citrobacter koseri (n = 1), Proteus mirabilis (n = 1), Morganella morganii (n = 1), Aspergillus spp. (n = 2), mould not further identified (n = 1), and acid-fast bacilli (n = 1).

Cultural sampling was driven by clinical suspicion rather than by a standardized surveillance protocol: at least one respiratory specimen was submitted for 178 of 189 POP patients (94.2%) but for only 121 of 316 non-POP patients (38.3%). Consequently, the absence of a positive culture does not establish the absence of bacterial pneumonia, pathogen frequencies describe only the culture-positive subgroup, and no comparison of pathogen distribution between POP and non-POP patients is made. The pathogen groups were small and differed in baseline severity (patients whose first isolate was S. aureus had lower WFNS grades and lower SAPS II), so the mortality figures are unadjusted and do not support pathogen-specific attributable mortality estimates.

Susceptibility results were available for 370 of the 865 respiratory isolates (42.8%). No susceptibility testing is recorded for yeast isolates. Among the 61 patients whose first isolate was S. aureus, oxacillin susceptibility was reported for 56: 11 were methicillin-resistant and 45 methicillin-susceptible; in-hospital mortality was 3 of 11 (27.3%) and 6 of 45 (13.3%), respectively (Fisher P = 0.358).
